# Single-cell transcriptomics reveals lipid metabolism reprogramming in macrophages in vitro during early stages of *Leishmania donovani* infection

**DOI:** 10.1186/s13071-026-07361-w

**Published:** 2026-03-13

**Authors:** Fei-Ran Li, Qi Sun, Qing Wang, Ling-Lin Qu, Zhi-Hui Li, Zheng-Jun Yi, Bin-Bin Yang

**Affiliations:** 1 School of Medical Laboratory, Shandong Second Medical University, Weifang, 261053 Shandong China; 2 Advanced Academy Engineering Research Institute for Precision Medicine Innovation and Transformation of Infectious Disease, Weifang, 261053 Shandong China; 3 People’s Hospital of Rongcheng, Weihai, 264300 Shandong China; 4 Heze Municipal Hospital, Heze, 274031 Shandong China

**Keywords:** Leishmaniasis, *Leishmania donovani*, Macrophages, Single-cell RNA sequencing, Infection mechanism

## Abstract

**Background:**

*Leishmania donovani* can cause visceral leishmaniasis, clinically manifested as fever, hepatosplenomegaly, and anemia. If left untreated, it may lead to death owing to complications from other diseases and infections. However, the pathogenesis of *L. donovani* infection remains unclear.

**Methods:**

In this study, we utilized single-cell transcriptomic sequencing technology to analyze the transcriptomic landscape of macrophages infected with *L. donovani* in mice.

**Results:**

The results showed that the parasite infection rate within macrophages gradually decreased over time. By comparing the transcriptomic profiles of infected cells, bystander cells, and unexposed cells, we identified precise modulation of several key pathways: first, the “*Fabp4*/*Cd36* lipid metabolism pathway” is activated during early infection. Significant changes in genes such as *Fabp4* are observed in infected cells, suggesting that the parasite hijacks the host lipid metabolic pathway to create a lipid sanctuary. Additionally, as the duration of infection increases, aging-related genes *Plk1*, *Cenpa*, *Bub1b*, *H2afx*, and *Cdkn2d* were activated, suggesting that infection may initiate cellular senescence. Meanwhile, infected cells mediated coordinated immune responses in bystander cells through secretory signals, as evidenced by concurrent expression trends of genes such as *Il1rn*, *Ccl3*, and *Hmox1* in both cell types. Further analysis revealed that parasite gene expression levels were significantly higher in M2 macrophages than in M1 macrophages, indicating that an M2-polarized microenvironment is more conducive to intracellular parasite survival. Additionally, parasite gene expression profiling revealed transcriptional remodeling characteristics during the transition from promastigotes to amastigotes, manifested as a significant decrease in the total number of expressed genes over time.

**Conclusions:**

This study analyzed the host immune response process and the characteristics of parasite–host interactions during *L. donovani* infection, which may provide a theoretical basis for the development of novel anti-infective treatment strategies in the future.

**Graphical Abstract:**

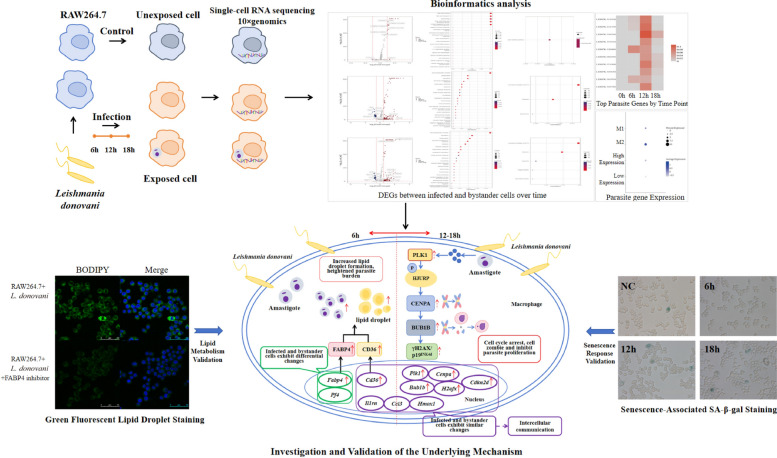

**Supplementary Information:**

The online version contains supplementary material available at 10.1186/s13071-026-07361-w.

## Background

Leishmaniasis is endemic in 100 countries and regions worldwide, with an annual incidence of 700,000 to 1 million new cases [1]. Depending on clinical manifestations, leishmaniasis is classified into visceral leishmaniasis (VL), cutaneous leishmaniasis, and mucocutaneous leishmaniasis [2]. VL, also known as kala-azar, is the second leading cause of death from parasitic diseases globally. VL is a zoonotic disease transmitted through the bite of infected sand flies, spreading between animal hosts (such as infected dogs) and humans or between humans. *Leishmania donovani* and *Leishmania infantum* are the pathogens of the disease, with *L. donovani* primarily prevalent in East Africa and South Asia [3]. Currently, the common treatment for VL is pathogen-directed therapy, utilizing drugs such as pentavalent antimonials and amphotericin B [4]. However, the development of emerging therapeutic drugs has been hindered owing to the unclear mechanism of infection.

*Leishmania donovani* is a unicellular protozoan parasite. Its life cycle comprises two stages: the promastigote stage residing within the sand fly’s digestive tract and the amastigote stage within the mammalian host. Promastigotes enter the mammalian host during the vector’s blood meal. Upon entry into the host, promastigotes are phagocytosed by macrophages, where they transform into the intracellular amastigote stage. The amastigotes are adapted to the intracellular environment, where they replicate and are responsible for the pathogenesis of the disease [5]. *Leishmania* parasites evade the host’s defense mechanisms by regulating their surface proteins or modulating host gene expression to suppress the host’s immune response. Studies have shown that altering epigenetic histone modifications plays a crucial role in chromatin remodeling in parasite [6–8]. However, the molecular mechanisms by which parasites induce epigenetic changes in the host remain to be elucidated.

After *L. donovani* enters macrophages, the parasite can proliferate intracellularly, establishing macrophages as its primary host cells. At the same time, macrophages also serve as the key effector cells in the anti-leishmanial immune response, responsible for parasite recognition and clearance [9, 10]. The plasticity of macrophages is a defining characteristic, enabling them to adapt to microenvironmental stimuli and mount functional responses [11, 12]. In *Leishmania* infection, this plasticity is exploited by the parasite to induce a shift in host cells from a killing state to a permissive state. This transition involves reciprocal regulation of cytokines, typically triggered by proinflammatory signals that subsequently induce anti-inflammatory factors, establishing a dynamic equilibrium conducive to parasite survival. Epigenetic alterations, changes in transcription factor activity, and regulatory RNA networks participate in modulating this reciprocal process during *Leishmania* infection [13]. Specifically, following infection, macrophages can be activated to produce interferon-γ (IFN-γ), tumor necrosis factor-α (TNF-α), and nitric oxide (NO), which are crucial for eliminating the parasite. Correspondingly, *L. donovani* and other *Leishmania* species have evolved multiple counterstrategies, including interfering with cytokine signaling, disrupting cellular metabolic pathways, and inducing the production of immunosuppressive molecules, thereby promoting their intracellular survival [14]. However, a comprehensive understanding of the specific interaction networks and infection dynamics between *L. donovani* and macrophages remains elusive. Single-cell transcriptomics provides a powerful tool for studying these interactions at the gene expression level, enabling precise characterization of heterogeneous cellular responses during infection.

Single-cell RNA sequencing (scRNA-seq) studies gene expression levels at the single-cell resolution, which helps to understand cellular heterogeneity and functional diversity [15]. It has played a significant role in research areas such as developmental biology, cancer biology, pathogenic microbial infection, and cellular homeostasis [16]. In recent years, scRNA-seq has demonstrated remarkable potential in the field of pathogenic microbial infection. For example, Lee et al. utilized scRNA-seq to characterize dermal TRMs in *Leishmania major* infected skin, and identify an unappreciated type 2 circuitry involving eosinophils and ILC2s, and orchestrated by MHCII-MR^high^ dermal TRMs [17]. Currently, scRNA-seq is divided into six main steps: preparation of live single-cell suspension, assessment of cell viability, removal of lysed cells, individual transcriptome barcoding, cDNA generation, and sequencing library generation [18]. In studying the gene expression characteristics of macrophages, scRNA-seq mainly focuses on exploring their immunopathogenesis, clinical progression, and treatment [14].

In this study, we employed scRNA-seq to monitor the intracellular gene expression changes in macrophages infected with *L. donovani* across different time points. We aimed to elucidate the variations in the infection rate of the parasite within macrophages under different time effects. Following the parasite infection, we defined and interpreted distinct cell clusters on the basis of various markers, ultimately conducting a comprehensive analysis of the mechanism by which the parasite infects macrophages.

## Methods

### *L. donovani* infection of RAW264.7 cells

The experimental groups were divided into a cell negative control group (RAW264.7 cells), a parasite control group (*L. donovani*), and experimental groups (*L. donovani*-infected cells at 6, 12, and 18 h). The parasite suspension was aspirated from the culture flask into a centrifuge tube. It was centrifuged at 3500 rpm for 5 min, and the supernatant was discarded. The pellet was resuspended in phosphate-buffered saline (PBS) and centrifuged again. This washing procedure was repeated three times. After washing, an appropriate amount of the parasite suspension was aspirated and evenly dropped onto a confocal fluorescence glass dish, which was then allowed to dry naturally at room temperature. RAW264.7 cells were prepared into a cell suspension at a density of 2 × 10^5^ cells/mL, and 2 mL of this suspension was evenly seeded into the confocal fluorescence glass dish. The dish was then incubated overnight at 37 ℃ with 5% CO_2_ in an incubator. Subsequently, the parasite suspension was prepared, and the parasites were used to infect the cells at a ratio of 20:1. The parasite suspension was collected and counted under a microscope. After centrifugation and washing, *L. donovani* were then resuspended in Dulbecco’s modified Eagle medium (DMEM) medium. Subsequently, 2 mL of the parasite suspension was added to the dish for infection periods of 6, 12, and 18 h, respectively. After infection, the dish was washed three times with 1 mL of PBS. Subsequently, the cells were then fixed with 4% paraformaldehyde and permeabilized with 0.4% Triton X-100, followed by washing. Then, 600 μL of Dil staining working solution (prepared according to the manufacturer’s instructions at a 1:250 dilution using the Servicebio^®^ Dil Cell Membrane Red Fluorescence Staining Kit, cat. no. G1705) was added and incubated in the dark at 37 ℃ for 20 min. After washing, an antifluorescence quenching mounting medium (with DAPI, Servicebio^®^, cat. no. G1407) was applied, and images were acquired using a Leica TCS SP8 laser confocal microscope.

### Sequencing sample preparation

Four samples were submitted for scRNA-seq. Mouse RAW264.7 cells were infected with stationary phase *L. donovani* promastigotes at a ratio of 20:1 and incubated for 6, 12, and 18 h. After incubation, the cells were washed three times with complete medium consisting of DMEM/high glucose, 10% fetal bovine serum (FBS), and 1% penicillin–streptomycin liquid (100×) to remove unattached promastigotes, and the cell culture flasks were filled with DMEM complete medium. The negative control used in this experiment was uninfected RAW264.7 cells. The samples were submitted to Beijing SeekGene BioSciences Co., Ltd. for single-cell library construction and sequencing.

### Cell preparation

After harvesting the samples, they were washed with ice-cold PBS (Hyclone SH30256.01) and dissociated using trypsin (Sigma SH30042.01). DNase I (Sigma 9003-98-9) treatment was applied optionally depending on the viscosity of the homogenate. Cell counting and viability detection were performed using the SeekMate Tinitan fluorescence cell counter (SeekGene M002C) in combination with AO/PI reagents (Miltenyi 130-109-398/130-090-101). Finally, the fresh cells were washed twice with RPMI1640 (Gibco 11875119) and then resuspended at a density of 1 × 10^6^ cells/mL in RPMI1640 supplemented with 2% fetal bovine serum (Gibco 10100147C).

### Single cell RNA-seq library construction and sequencing

Single-cell RNA-Seq libraries were prepared using the SeekOne^®^ DD Single Cell 3′ library preparation kit (SeekGene, cat. no. K00202). Briefly, a range of 20,000–22,000 cells were mixed with reverse transcription reagents and then loaded into the sample well of a SeekOne^®^ chip S3. Subsequently, barcoded hydrogel beads (BHBs) and partitioning oil were dispensed into their corresponding wells separately in chip S3. After emulsion droplet generation, reverse transcription was performed at 42 ℃ for 90 min and inactivated at 85 ℃ for 5 min. Next, complementary DNA (cDNA) was purified from broken droplets and amplified by PCR under the following conditions: 98 ℃ for 3 min; 13 cycles of 98 ℃ for 10 s, 63 ℃ for 15 s, and 72 ℃ for 3 min, followed by 72 ℃ for 5 min and hold at 4 ℃. The amplified cDNA product was then purified using VAHTS DNA Clean Beads (Vazyme N411-01) and subsequently fragmented, end-repaired, A-tailed, and ligated to sequencing adapters using the Illumina kit (cat. no. 20104706). Finally, the indexed PCR were performed to amplify the DNA fragments representing the 3′ polyA regions of expressing genes, which also contained cell barcode and unique molecular index. The indexed sequencing libraries were purified using VAHTS DNA Clean Beads and assessed with a Qubit (Thermo Fisher Scientific Q33226) and a Bio-Fragment Analyzer (Bioptic Qsep400). The libraries were then sequenced on an Illumina NovaSeq X Plus platform with PE150 read length.

### Raw data processing

Raw sequencing data (FASTQ files) were processed using Cell Ranger (version 9.0.0), the official software from 10x Genomics. After quality assessment, reads were aligned to reference genomes. Two reference genomes were used: *Mus musculus* genome GRCm39 (GCF_000001635.27) and *L. donovani* genome ASM22713v2 (GCA_000227135.2). Gene expression quantification results were obtained using the count module of Cell Ranger, generating a gene expression matrix for subsequent analysis.

### Dimension reduction and cluster analysis

Raw data underwent standard analysis using Seurat (version 4.1.1) [19]. Raw data were filtered according to the following parameters to achieve standard sample quality control [20]: 1. number of genes detected per cell (Feature.range: 100–30,000); 2. number of transcripts per cell (Umi.range: 200–100,000); 3. proportion of mitochondrial-related gene set expression (mitorelatedgenes.range: 0–20%).

To integrate samples from four infection time points and eliminate batch effects, we employed the Harmony algorithm (version 0.1) [21]. First, the variance-stabilizing transformation (VST) method was used to identify 2,000 highly variable genes in the integrated dataset. Subsequently, principal component analysis (PCA) was performed based on these genes. Set the resolution (default 0.5) for cell clustering to adjust the number of clusters. On the basis of the number of PCA components corresponding to the PCA inflection point, we set the dimensionality reduction dimension used for clustering (default 30). This value typically ranges from 10 to 30, primarily determined by the biological complexity of the samples.

### Differential enrichment analysis

We conducted a differential expression analysis across multiple groups, utilizing RNA assays, which involved raw data without batching or normalization. The data were analyzed using the Wilcoxon rank-sum test, a method that does not require the assumption of a normal distribution for the overall data, to determine whether the overall distributions of the two groups are identical. The minimum gene expression ratio was set at 0.1, or 10%. The screening threshold for differentially expressed genes was |fold change| ≥ 1.2, equivalent to |log_2_FC| > 0.263. Raw *P*-values were adjusted for multiple hypothesis testing using the Benjamini–Hochberg method to control the false discovery rate (FDR), and the adjusted *P*-value (i.e., FDR) threshold was set to < 0.05. Differentially expressed genes were analyzed using the R package ClusterProfiler (version 4.2.0) [22] for Gene Ontology (GO) and Kyoto Encyclopedia of Genes and Genomes (KEGG) enrichment analysis. The results were visualized using ggplot2 (version 3.3.6) [23] in R. Overlap representation analysis (ORA) was employed for enrichment analysis. Studies have shown that variations in parameters such as the background set, differential metabolite selection method, and the pathway database used can lead to differences in ORA results [24]. We set the screening threshold for *P*-value cutoff (i.e., determining whether the enrichment analysis results are significant) to 0.05, and the screening threshold for *q*-value cutoff (i.e., the corrected *P*-value) to 0.05. The gene set size range is between 10 and 500.

### Protein interaction networks and hub gene analysis

To analyze functional associations among differentially expressed genes, we constructed a protein interaction network on the basis of the STRING database. This database systematically evaluates functional relationships between proteins by integrating multiple evidence sources—including experimental validation, coexpression, text mining, and known pathway knowledge—and provides a comprehensive confidence score [25]. The specific workflow is as follows: the top 200 significantly differentially expressed genes from each comparison group were submitted to the STRING online platform, with the species set to “*Mus musculus*”. To select high-confidence interactions, only interaction pairs with a composite confidence score > 0.4 were retained. The obtained network data (including nodes, edges, and scores) were imported into Cytoscape software (v3.10.2) for visualization and further analysis. Proteins, namely differentially expressed genes, were represented by nodes, and interactions between two proteins were represented by edges [26]. Cytoscape performed a simulated annealing algorithm on the initial network imported from the raw data, providing a new elastic network in which loosely connected edges were filtered and removed [27]. Subsequently, the CytoHubba plugin in Cytoscape was employed to identify hub genes in the network using both the degree and maximum cluster centricity (MCC) algorithms. The top ten genes ranked by each algorithm were selected for subsequent biological interpretation.

### Western blot verification

We infected RAW264.7 cells with *L. donovani* promastigotes at a ratio of 20:1 and incubated them for 6, 12, and 18 h. The negative control group consisted of uninfected RAW264.7 cells. After incubation, cells were lysed using radioimmunoprecipitation assay (RIPA) and phenylmethylsulfonyl fluoride (PMSF) at a ratio of 100:1 to extract proteins. Subsequently, proteins from each group were loaded at a mass of 20 μg. After electrophoresis, the membrane was transferred. After blocking, the membranes were incubated overnight at 4 ℃ with the following primary antibodies: rabbit anti-heme oxygenase 1 (HMOX1) polyclonal antibody (1:700 dilution), rabbit anti-macrophage inflammatory protein 1 alpha/CCL3 polyclonal antibody (1:400 dilution), rabbit anti-IL-1RA (IL-1RN) polyclonal antibody (1:700 dilution), and rabbit anti-GAPDH polyclonal antibody (1:10,000 dilution). After washing, the membranes were incubated with horseradish peroxidase (HRP)-conjugated Affinipure Goat Anti-Rabbit IgG (H + L) secondary antibody (1:5,000 dilution) for 1 h at room temperature with gentle shaking. Finally, images were captured using a Bio-Rad UV gel imaging system (settings: Blot-Chemi, no filter).

### Immunofluorescence validation

Mice RAW264.7 cells were infected with stationary phase *L. donovani* promastigotes at a 20:1 ratio and incubated for 6, 12, and 18 h. The negative control group consisted of uninfected RAW264.7 cells. After infection, cells were fixed with 4% paraformaldehyde for 30 min at room temperature, followed by permeabilization with 0.4% Triton X-100 for 30 min at room temperature. Blocking was performed with 5% bovine serum albumin (BSA) for 30 min at 37 ℃. The cells were then incubated overnight at 4 ℃ in a humidified chamber with primary antibodies: rabbit anti-IL-1RN polyclonal antibody (1:500 dilution), rabbit anti-CCL3 polyclonal antibody (1:1000 dilution), rabbit anti-HMOX1 polyclonal antibody (1:600 dilution), and rabbit anti-FABP4 polyclonal antibody (1:500 dilution). Then, cells were washed three times with PBS (5 min each). Subsequently, cells were incubated with fluorescein isothiocyanate (FITC)-conjugated goat anti-rabbit IgG (H + L) secondary antibody (1:500 dilution) for 1 h at 37 ℃ in the dark. After washing three times with PBS (5 min each), images were acquired using a Leica TCS SP8 confocal laser scanning microscope.

### β-Galactosidase senescence staining

Mice RAW264.7 cells were infected with stationary phase *L. donovani* promastigotes at a 20:1 ratio and incubated for 6, 12, and 18 h. The negative control group consisted of uninfected RAW264.7 cells. Following infection, cells were stained using the β-galactosidase staining kit (Solarbio, G1580) according to the manufacturer’s instructions. Images were captured under a light microscope after staining completion.

### Cell counting kit 8 (CCK-8) assay

The cytotoxic effects of the FABP4 inhibitor (BMS309403) (Selleck, S6622) and the CD36 inhibitor (sulfosuccinimidyl oleate sodium, SSO) (Targetmol, T13036L) on RAW264.7 cells were evaluated using the CCK-8 assay. In total, 8,000 cells were seeded per well in 96-well plates. After cell attachment, inhibitor treatment groups were added with 40 μM BMS309403 and 50 μM SSO, respectively. Following a 24-h incubation, 10 μL of CCK-8 reagent was added, and absorbance at 450 nm was measured using a full-wavelength microplate reader.

### Lipid droplet staining

The experimental groups were as follows: the negative control group (RAW264.7 cells), the positive control group (*L. donovani* promastigotes in the stationary phase infected cells at a ratio of 20:1 for 6 h), and the experimental group (FABP4/CD36 inhibitor + *L. donovani* infection for 6 h). The cells were seeded in confocal fluorescence glass dishes and cultured overnight. In one experimental group dish, the FABP4 inhibitor (BMS309403) was added at a concentration of 40 μM (Selleck, S6622), and in the other, the CD36 inhibitor (Sulfosuccinimidyl oleate sodium, SSO) was added at a concentration of 50 μM (Targetmol, T13036L). After 24 h of culture, the parasites were added for infection. The cells were washed with PBS and fixed, then stained with the lipid droplet green fluorescence detection kit (Beyotime, C2053S) according to the instructions. After staining, the cells were photographed under a confocal laser microscope.

### Data analysis

Data are presented as mean ± standard deviation. Intergroup differences were analyzed using one-way analysis of variance (ANOVA) with GraphPad Prism 8.0 software.

## Result

### Fluorescence staining and laser confocal identification

**Fig. 1:**
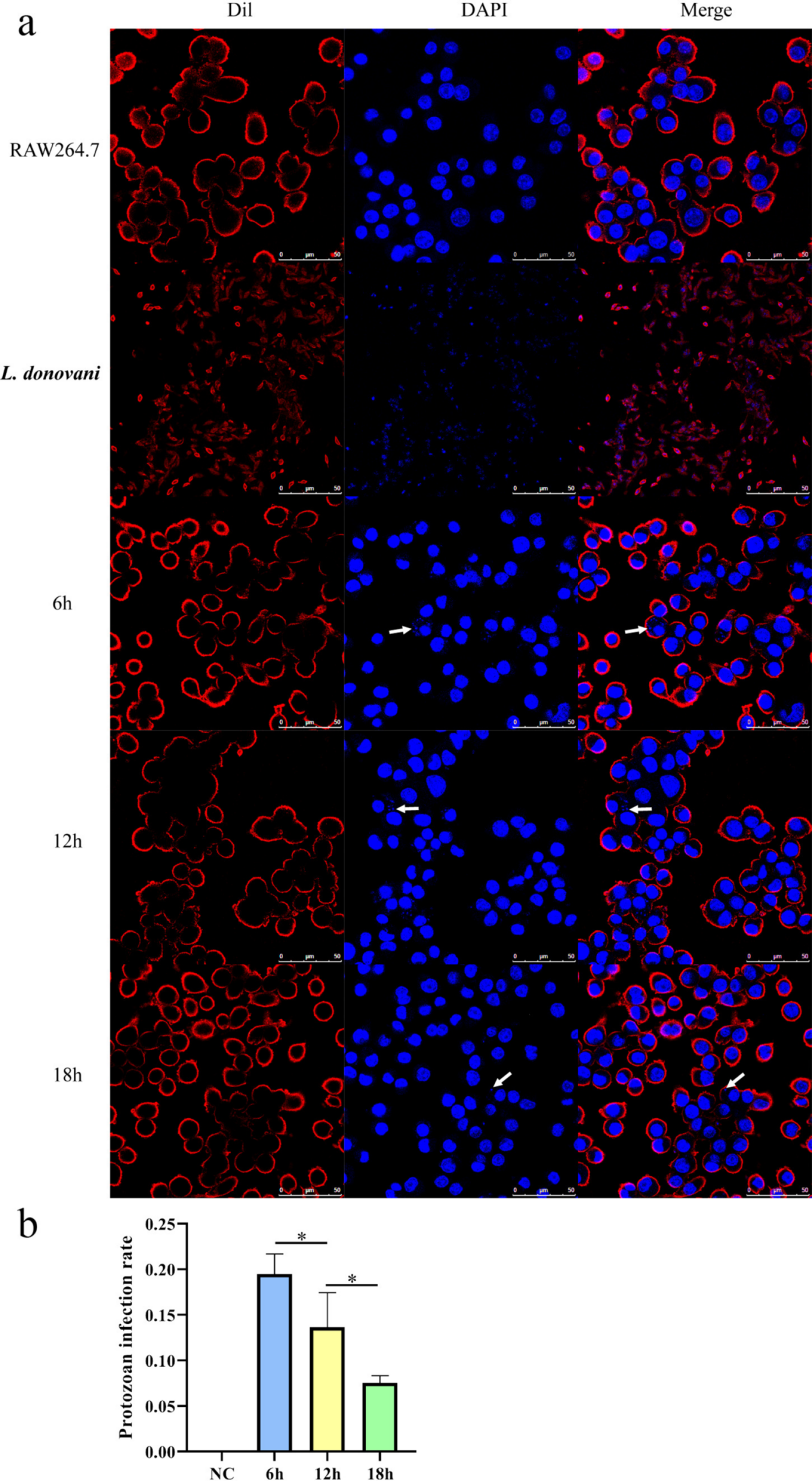
Fluorescence staining and laser confocal identification results of intracellular parasite infection at different time points. **a**, DAPI staining of macrophage and *L. donovani* nuclei, appearing as blue fluorescence; Dil staining of macrophage and *L. donovani* membranes, appearing as red fluorescence. White arrows indicate intracellular *L. donovani* parasites. **b**, Parasite infection rates at different time points. Data are expressed as mean ± standard deviation (*n* = 3 per group). *P < 0.05, **P < 0.01, *** *P* < 0.001. Statistical analysis was performed using ordinary one-way ANOVA followed by Tukey’s multiple comparisons test. The overall ANOVA result is significant (*F*_(3, 8)_ = 42.41, *P* < 0.0001). Adjusted *P*-values for the key comparisons are: 6 h versus 12 h, *P* = 0.0497; 12 h versus 18 h, *P* = 0.0395

Macrophages were infected with stationary-phase *L. donovani* promastigotes for 6, 12, and 18 h and then observed under a confocal laser microscope. Red fluorescence indicated the cell membrane, while blue fluorescence indicated the cell nucleus (Fig. 1a). The number of blue fluorescent signals from parasites nuclei within macrophages at different time points showed a gradual decrease, indicating a gradual reduction in the number of parasites infecting the cells. To quantitatively assess infection efficiency at different time points, we calculated the infection rate (the percentage of cells containing parasites relative to the total number of cells counted). The procedure was repeated three times for statistical analysis. Quantitative analysis revealed that the infection rate exhibited a gradual decline over time (Fig. 1b).

### Single-cell RNA sequencing reveals distinct macrophage clusters after *L. donovani* infection

A total of 10,534 cells were captured from uninfected samples, 10,768 cells from 6-h infected samples, 11,601 cells from 12-h infected samples, and 14,649 cells from 18-h infected samples. Cells were filtered on the basis of RNA abundance threshold, gene expression number threshold, and gene set expression ratio threshold, resulting in 10,322 cells from uninfected samples, 10,391 cells from 6-h infected samples, 11,205 cells from 12-h infected samples, and 14,233 cells from 18-h infected samples (Supplementary Table 1).

**Fig. 2:**
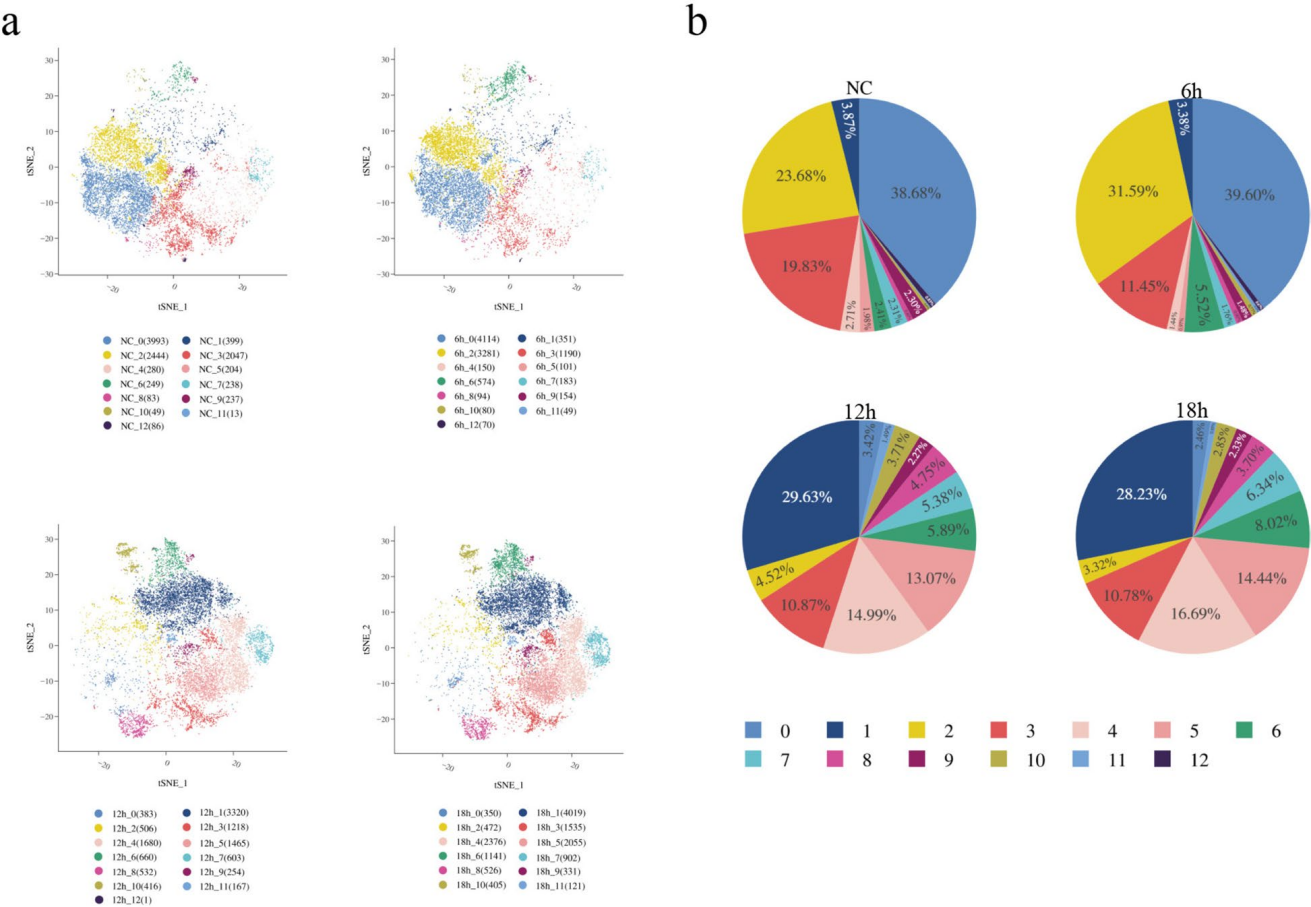
Transcriptome analysis results of cell samples at different infection time points. **a**, Dimensionality reduction plots of cell cluster distribution for the negative control group, 6-h sample group, 12-h sample group, and 18-h sample group. The numbers in parentheses indicate the number of cells contained in each cell cluster. **b**, Pie chart of cell cluster proportion. Colored squares represent cell clusters

Using the VST method to select hypervariable genes, the Harmony algorithm was employed to integrate the cells, followed by dimensionality reduction using PCA. After clustering the cells, 13 cell clusters were observed in the negative control, 6-h, and 12-h samples, while 12 clusters were observed in the 18-h sample (Fig. 2a). The cell counts within these clusters varied, and the percentage distribution of cell clusters among the total cells differed (Fig. 2b).

To analyze macrophage heterogeneity during infection, we identified multiple distinct macrophage subpopulations with well-defined functional characteristics by analyzing differentially expressed genes across cell clusters (Supplementary Table 2). By comparing conserved marker genes across different time points among cell clusters, cell clusters with the same number exhibit stable core transcriptional characteristics at different time points.

Among these, clusters 4 and 7 consistently overexpressed cell cycle-related genes (e.g., *Mki67*,* Top2a*, *Ube2c*) at all time points and were defined as proliferative macrophage subpopulations. Cluster 11 consistently overexpressed interferon-stimulated genes (e.g., *Isg15*), representing an interferon-responsive subpopulation. Post infection (particularly at 12 h and 18 h), clusters 0, 2, and 3 exhibited significant upregulation of chemokines (e.g., *Spp1*, *Ccl2/3/4*), suggesting proinflammatory recruitment functions. Additionally, cluster 8 exhibits distinct characteristics, with marker genes including complement components *Clqa/b/c* and* Pf4*. At 6 h post infection, the key lipid metabolism gene *Fabp4* is upregulated in this cluster, defining it as a unique “high-*Fabp4*-expressing complement-associated macrophage subpopulation.”

### Analysis of the impact of parasite infection duration on the infection rate within macrophages

**Fig. 3:**
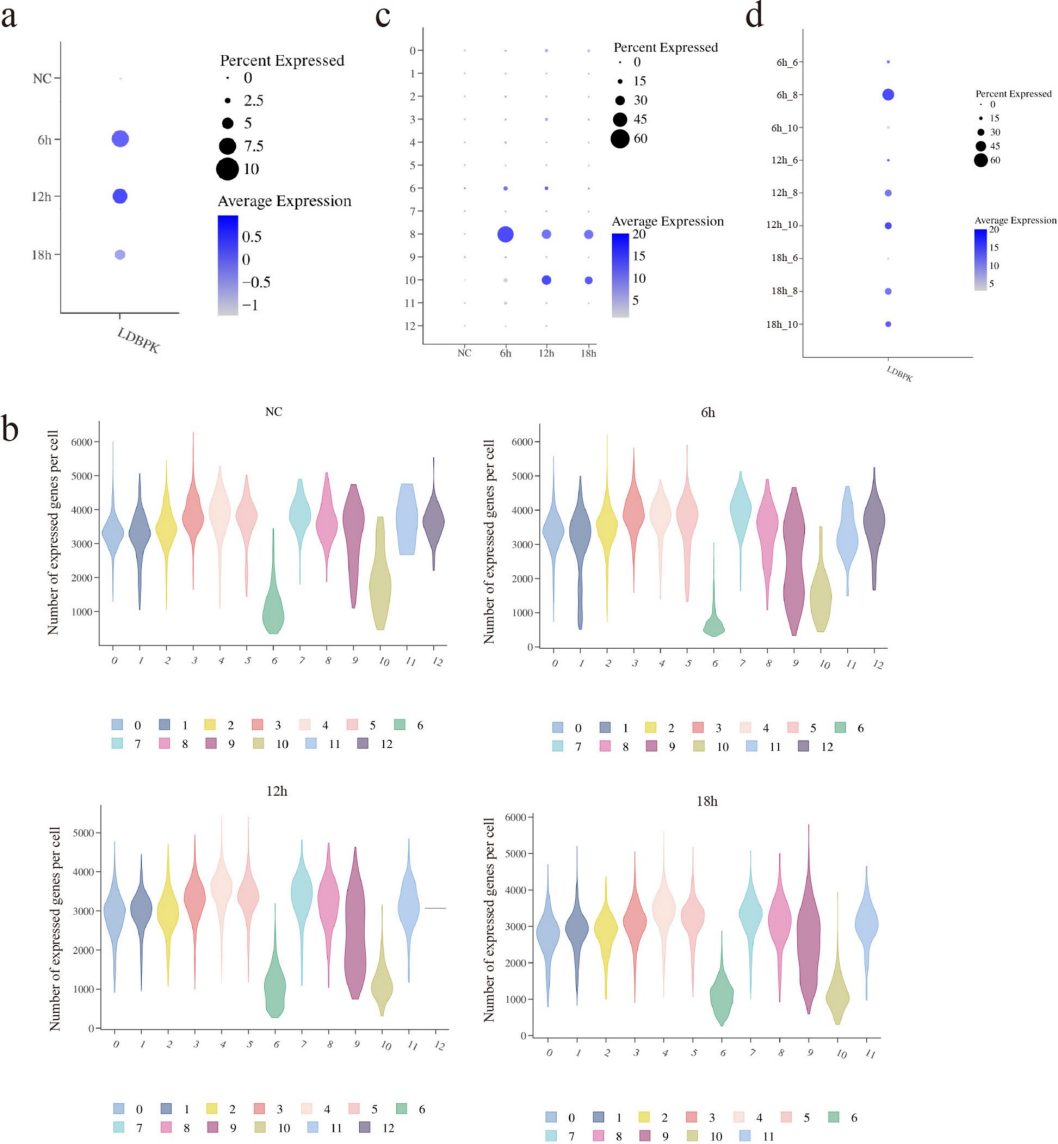
Analysis of the effect of parasites infection duration on infection rates within macrophages. **a**, A bubble chart displays the expression of the LDBPK gene set in macrophages across different time points. Bubble size indicates the percentage of cells expressing LDBPK within each group; color intensity reflects the average expression level of the gene. **b**, Violin plots displaying the distribution of total gene counts per cell (number of expressed genes) for each macrophage cluster at the four time points. Each cluster is shown separately; the width of the violin reflects cell density. **c**, The merged bubble plot displays the expression level of LDBPK signature genes across different cell clusters (*y*-axis) and cell groups at different time points (*x*-axis). Bubble size and color correspond to the definitions in panel **a**. Cluster 12 was not detected in the 18-h samples, hence the empty space at that position. **d**, Bubble plot focusing on clusters 6, 8, and 10, showing LDBPK expression across time points

To assess infection dynamics, we constructed a comprehensive feature (termed “LDBPK,” named after the common prefix of all detected *L. donovani* gene IDs) on the basis of parasite genes detected in the complete *L. donovani* reference genome (assembly version ASM22713v2) to quantify the parasite transcriptional load within each cell. This feature is computed using the AddModuleScore function from the Seurat package. Its principle involves generating a standardized enrichment score for each cell by comparing the target gene set (i.e., all detected *L. donovani* genes, which we named “LDBPK”) with a set of background control genes exhibiting comparable expression levels. We hypothesize that the more active and abundant the live parasites are within infected cells, the stronger their transcriptional activity will be, manifesting as higher LDBPK scores. Thus, detecting this feature via scRNA-seq indirectly reflects infection levels. In scRNA-seq analysis of macrophages infected with *L. donovani* at 6, 12, and 18 h, the results showed that as time progressed, the proportion of cells expressing the parasite gene set gradually decreased, with 0% in uninfected samples, 7.47% at 6 h, 6.55% at 12 h, and 4.53% at 18 h, indicating that the parasite infection rate in macrophages gradually decreased with time (Fig. 3a), consistent with the trend of fluorescence staining results. Additionally, when analyzing the distribution of gene expression levels across various macrophage clusters, we found that compared with other clusters, most cells in clusters 6 and 10 exhibited lower gene expression levels (Fig. 3b). Further analysis of the changes in parasite infection rates across different macrophage clusters revealed that compared with other clusters, clusters 6, 8, and 10 showed more significant changes in parasite infection rates (Supplementary Table 3, Fig. 3c and d).

### Temporal transcriptomic dynamics analysis of *L. donovani*-infected macrophages

#### Differentially expressed gene screening and functional enrichment analysis

On the basis of different infection time gradients, we conducted differential expression analysis among multiple groups. Differentially expressed genes (DEGs) were screened using the criteria described above (|fold change| ≥ 1.2, i.e., |log_2_FC| > 0.263 and adjusted *P*-value < 0.05), followed by GO and KEGG functional enrichment analysis. The analysis results are presented in the table (Supplementary Table 4).

**Fig. 4:**
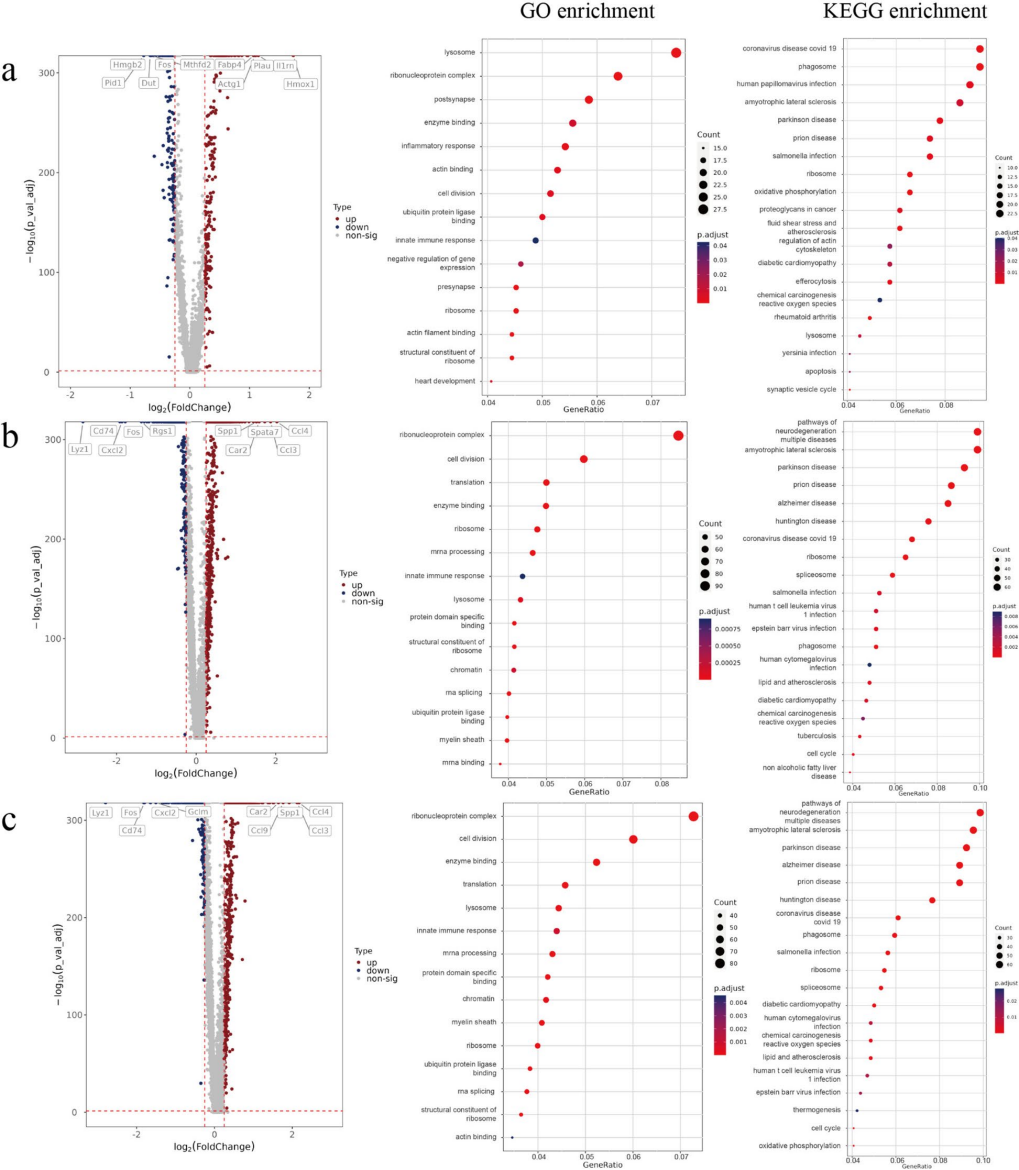
Transcriptomic profiling and functional enrichment analysis of differentially expressed genes (DEGs). **a**–**c**, Volcano plots and GO/KEGG enrichment bubble plots of DEGs for the **a** 6 h, **b** 12 h, and **c** 18 h sample groups compared with the negative control. Results for comparisons between infected time points (12 h versus 6 h, 18 h versus 6 h, and 18 h versus 12 h) are shown in Supplementary Fig. 1

After comparing the 6-h sample group with the negative control group, a total of 355 DEGs were identified, with 203 genes upregulated and 152 genes downregulated. GO and KEGG enrichment analysis results are shown in the figure (Fig. 4a).

After comparing the 12-h sample group with the negative control group, a total of 1,108 DEGs were identified, with 662 genes upregulated and 446 genes downregulated. GO and KEGG enrichment analysis results are shown in the figure (Fig. 4b).

After comparing the 18-h sample group with the negative control group, a total of 1,136 DEGs were identified, with 663 genes upregulated and 473 genes downregulated. GO and KEGG enrichment analysis results are shown in the figure (Fig. 4c).

After comparing the 12-h sample group with the 6-h sample group, a total of 1421 DEGs were identified, of which 861 genes were upregulated and 560 genes were downregulated. GO and KEGG enrichment analysis results are shown in the figure (Supplementary Fig. 1a).

After comparing the 18-h sample group with the 6-h sample group, a total of 1,464 DEGs were identified, of which 860 genes were upregulated and 604 genes were downregulated. GO and KEGG enrichment analysis results are shown in the figure (Supplementary Fig. 1b).

After comparing the 18-h sample group with the 12-h sample group, a total of 17 DEGs were identified, with eight genes upregulated and nine genes downregulated. GO and KEGG enrichment analysis results are shown in the figure (Supplementary Fig. 1c). Violin plots of the comparative results for differentially expressed genes across the above groups, along with UMAP plots mapping their expression levels, are provided in Additional file 1 (Supplementary Figs. 2–3).

After conducting a comprehensive comparative analysis of samples collected at different infection time points against negative control samples, the results revealed that several DEGs in key functional pathways exhibited significant changes. These alterations contribute to understanding parasite survival and host anti-infection mechanisms. The expression of *Fabp4* and *Cd36*, related to lipid metabolism pathways, peaked at 6 h and then gradually decreased. The expression of *Plk1*, *Cenpa*, *Bub1b*, *H2afx*, and *Cdkn2d*, associated with aging pathways, showed time-dependent upregulation (Supplementary Table 5).

#### Identification of core modules in protein–protein interaction networks

On the basis of the temporal functional enrichment characteristics of the aforementioned DEGs, to further reveal the synergistic mechanism of key regulatory factors during the infection process, we further analyzed the hub genes interacting with DEGs by constructing a protein–protein interaction network.

**Fig. 5:**
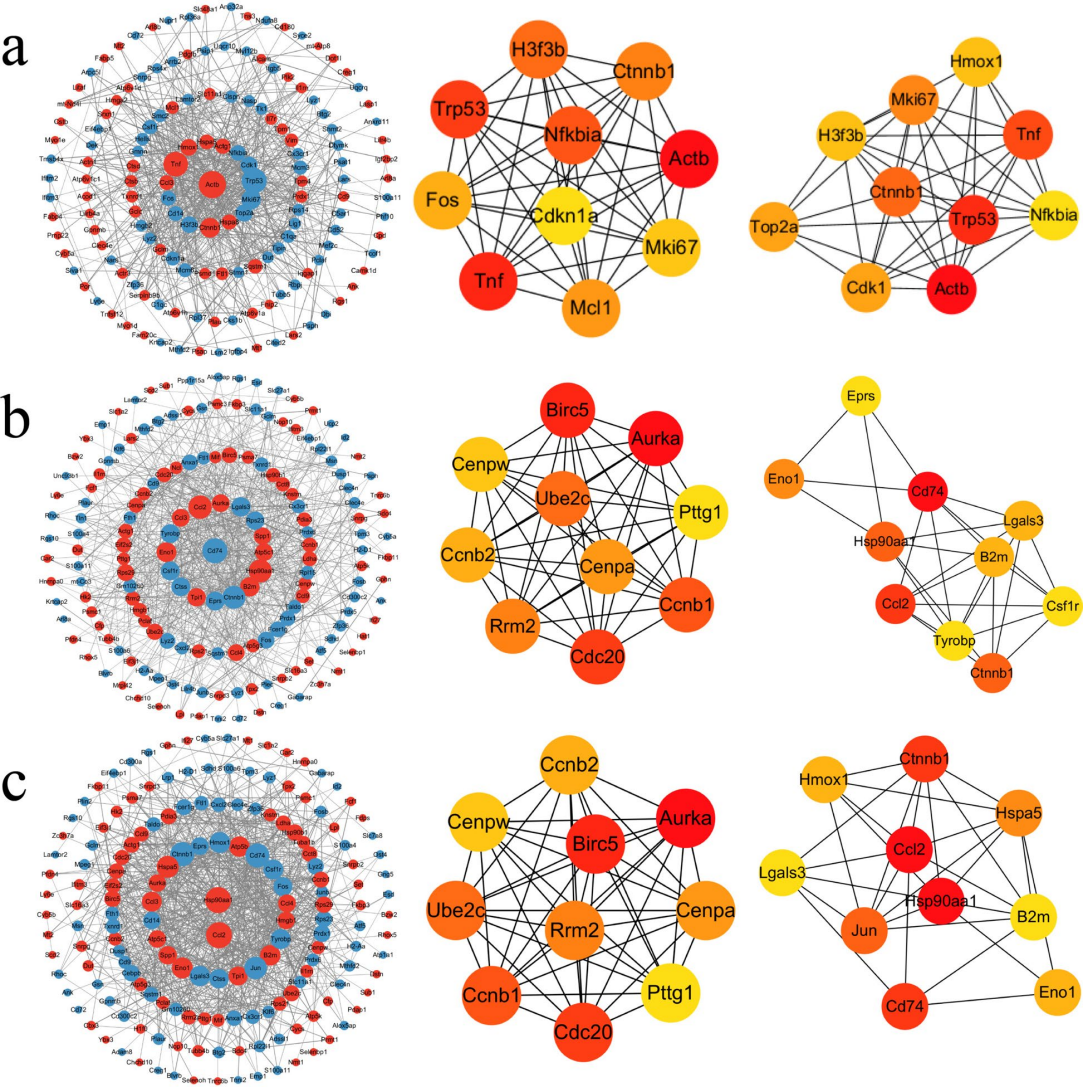
Protein–protein interaction networks and hub genes of top differentially expressed genes. PPI networks were constructed using the top 200 DEGs from each pairwise comparison. Within each network, node colors indicate differential expression: red for upregulated and blue for downregulated genes. Hub genes within each network were identified using the CytoHubba plugin for Cytoscape. From left to right: PPI networks, top ten hub genes identified by MCC and Degree algorithms. **a**, 6 h versus NC. **b**, 12 h versus NC. **c**, 18 h versus NC. Results for comparisons between infected time points (12 h versus 6 h, 18 h versus 6 h, and 18 h versus 12 h) are shown in Supplementary Fig. 4

After comparing the 6-h sample group with the negative control group, the protein–protein interaction (PPI) network of the top 200 DEGs consisted of 153 nodes and 679 edges. CytoHubba identified the top ten hub genes in the network. The hub genes obtained by the MCC algorithm were *Actb, Tnf*, *Trp53*, *Nfkbia*, *H3f3b*, *Ctnnb1*, *Mcl1*, *Fos*, *Mki67*, and *Cdkn1a*. The hub genes obtained by the Degree algorithm were *Actb*, *Trp53*, *Tnf*, *Ctnnb1*, *Mki67*, *Top2a*, *Cdk1*, *H3f3b*, *Hmox1*, and *Nfkbia* (Fig. 5a).

After comparing the 12-h sample group with the negative control group, the PPI network of the top 200 DEGs consisted of 165 nodes and 739 edges. CytoHubba identified the top ten hub genes in the network, and the hub genes obtained by the MCC algorithm were *Aurka*, *Birc5*, *Cdc20*, *Ccnb1*, *Ube2c*, *Rrm2*, *Cenpa*, *Ccnb2*, *Cenpw*, and *Pttg1*. The hub genes obtained by the Degree algorithm were *Cd74*, *Ccl2*, *Ctnnb1*, *Hsp90aa1*, *Eno1*, *B2m*, *Lgals3*, *Eprs*, *Csf1r*, and *Tyrobp* (Fig. 5b).

After comparing the 18-h sample group with the negative control group, the PPI network of the top 200 DEGs was composed of 166 nodes and 835 edges. CytoHubba identified the top ten hub genes in the network, and the hub genes obtained by the MCC algorithm were *Aurka*, *Birc5*, *Cdc20*, *Ccnb1*, *Ube2c*, *Rrm2*, *Cenpa*, *Ccnb2*, *Cenpw*, and *Pttg1*. The hub genes obtained by the Degree algorithm were *Ccl2*, *Hsp90aa1*, *Cd74*, *Ctnnb1*, *Jun*, *Hspa5*, *Hmox1*, *Eno1*, *B2m*, and *Lgals3* (Fig. 5c).

After comparing the 12-h sample group with the 6-h sample group, the PPI network of the top 200 DEGs consists of 168 nodes and 913 edges. CytoHubba identified the top ten hub genes in the network, and the hub genes obtained by the MCC algorithm were *Birc5*, *Cdc20*, *Top2a*, *Rrm2*, *Cdca3*, *Cdk1*, *Ccnb1*, *Ccnb2*, *Cdca8*, and *Cenpa*. The hub genes obtained by the Degree algorithm were *Tnf*, *Ctnnb1*, *Ccl2*, *Cd74*, *Hsp90aa1*, *Birc5*, *Cdk1*, *Top2a*, *Aurka*, and *Anxa5* (Supplementary Fig. 4a).

After comparing the 18-h sample group with the 6-h sample group, the PPI network of the top 200 DEGs consists of 164 nodes and 846 edges. CytoHubba identified the top ten hub genes in the network, and the hub genes obtained by the MCC algorithm were *Birc5*, *Cdc20*, *Top2a*, *Rrm2*, *Cdca3*, *Cdk1*, *Ccnb1*, *Ccnb2*, *Cdca8*, and *Cenpa*. The hub genes obtained by the Degree algorithm were *Tnf*, *Ctnnb1*, *Ccl2*, *Cd74*, *Birc5*, *Top2a*, *Cdk1*, *Aurka*, *Cdca8*, and *Ccnb1* (Supplementary Fig. 4b).

After comparing the 18-h sample group with the 12-h sample group, the PPI network of 17 DEGs was composed of 11 nodes and eight edges. CytoHubba identified the top five hub genes in the network, and the hub genes obtained by the MCC algorithm were *Hmox1*, *Hspa5*, *H2ax*, *Glrx*, and *Spp1*. The hub genes obtained by the Degree algorithm were *Hmox1*, *Hspa5*, *H2ax*, *Glrx*, and *Spp1* (Supplementary Fig. 4c).

### Transcriptome dynamic analysis of macrophage subpopulations infected with *L. donovani*

#### Differential gene expression analysis and functional enrichment analysis of the overall transcriptome of infected or bystander cells

First, during the experimental design phase, the reference genome of *L. donovani* (ASM22713v2) was incorporated into the analysis workflow alongside the mouse genome to simultaneously capture transcripts from both host and pathogen. In data analysis, to quantify the parasitic transcriptional load per cell, we calculated a normalized composite score—the LDBPK feature value—using the AddModuleScore function from the Seurat software package, on the basis of all detected *L. donovani* genes. We designated cells from uninfected samples (negative controls) as unexposed cells, while cells from infected samples at different time points were classified as exposed cells. Exposed cells were further subdivided into infected cells and uninfected cells (bystander cells). Given that all cells in the uninfected control group exhibited LDBPK values of 0, this threshold was adopted as the background threshold. In infected samples, cells with LDBPK scores > 0 were classified as “infected cells”; otherwise, they were classified as “bystander cells.” Subsequently, using the same screening criteria, we identified DEGs between infected and bystander cells and performed enrichment analysis (Supplementary Table 6).

**Fig. 6:**
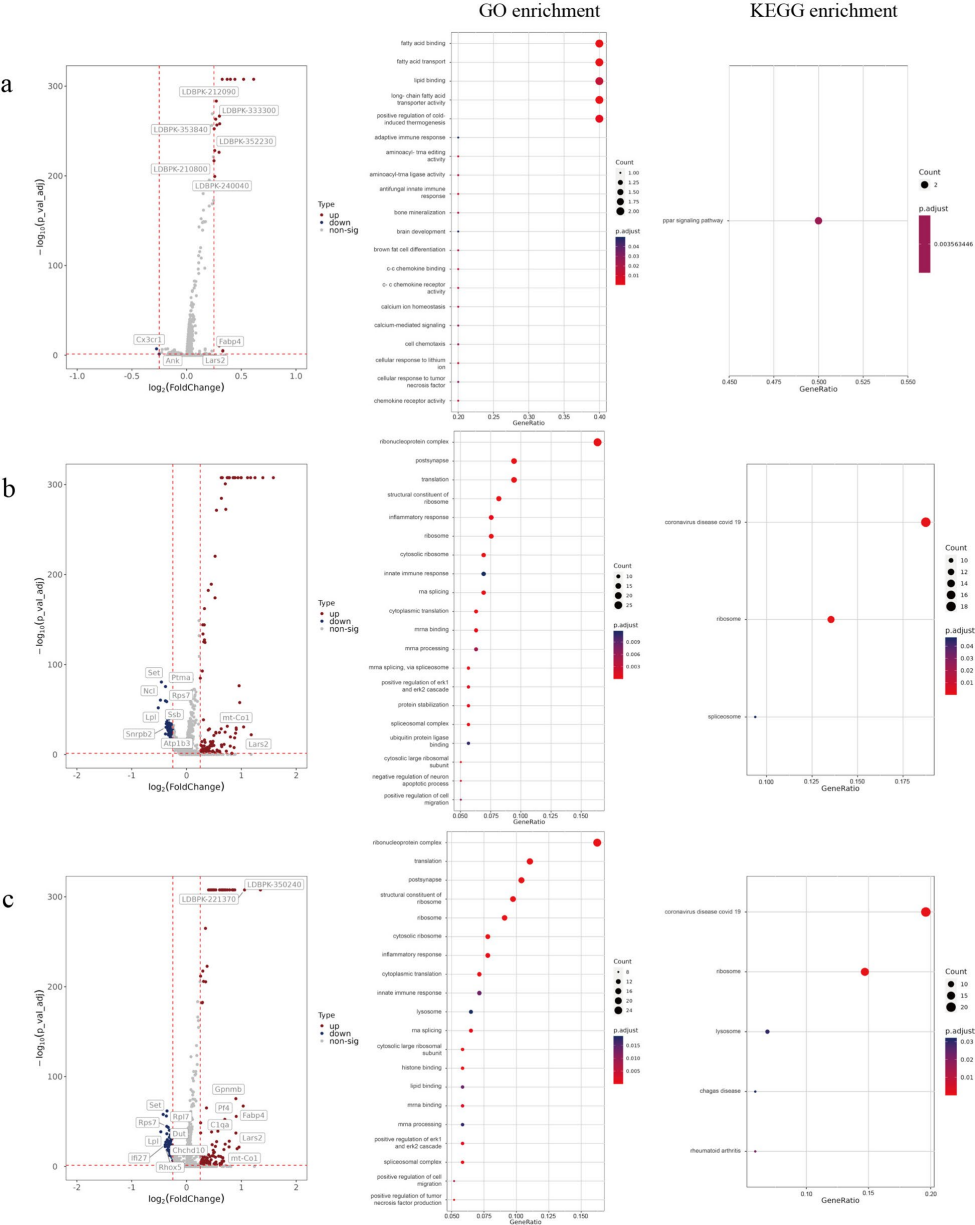
Comparison of infected cells and bystander cells at different time points. **a**–**c**, Volcano plots of DEGs in infected cells and bystander cells at 6, 12, and 18 h; GO and KEGG pathway enrichment bubble plots of DEGs

After comparing infected cells with bystander cells in the 6-h sample group, a total of 17 DEGs were identified, of which 16 genes were upregulated, and 1 gene was downregulated. GO and KEGG enrichment analysis results are shown in the figure (Fig. 6a).

After comparing infected cells with bystander cells in the 12-h sample group, a total of 193 DEGs were identified, including 116 upregulated genes and 77 downregulated genes. GO and KEGG enrichment analysis results are shown in the figure (Fig. 6b).

After comparing infected cells with bystander cells in the 18-h sample group, a total of 175 DEGs were identified, including 112 upregulated genes and 63 downregulated genes. GO and KEGG enrichment analysis results are shown in the figure (Fig. 6c).

Comparative analysis of infected cells and bystander cells at different infection time points revealed significant changes in several DEGs within key functional pathways. Expression levels of *Plk1*, *Cenpa*, *Bub1b*, *H2afx*, and *Cdkn2d*—associated with aging pathways—showed no significant differences between infected and bystander cells. By contrast, the expression of *Fabp4*, associated with lipid metabolism pathways, exhibited time-dependent upregulation (Supplementary Table 7).

##### Analysis of gene expression heterogeneity in infection time-dependent cell subpopulations

**Fig. 7:**
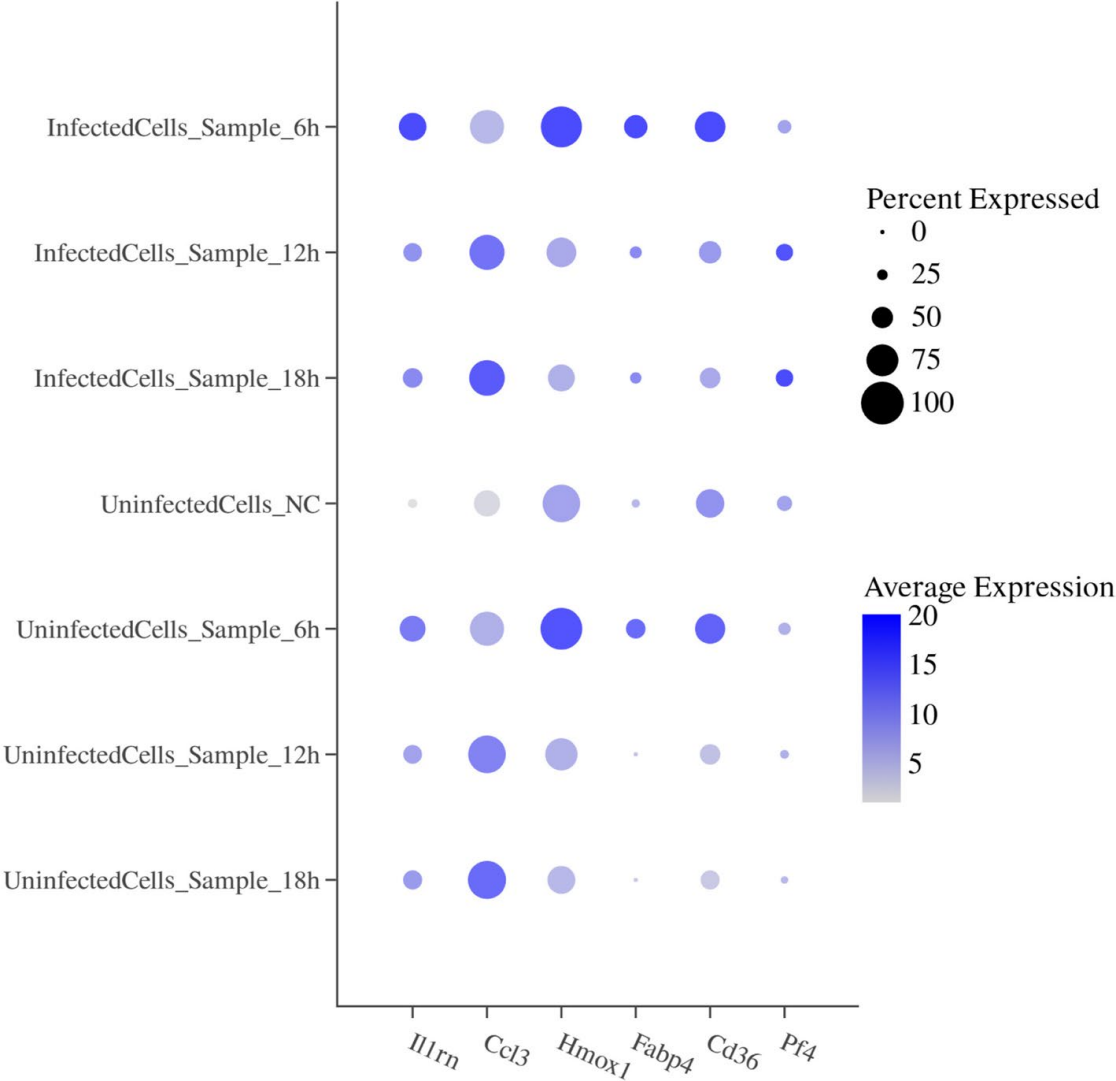
Expression of certain genes at different infection time points in unexposed cells, infected cells, and bystander cells. The *x*-axis represents six key genes: *Il1rn*, *Ccl3*, *Hmox1*, *Fabp4*, *Cd36*, and *Pf4*. The *y*-axis indicates grouping. Bubble color reflects the average gene expression level, with darker shades indicating higher average expression. Bubble size represents the expression percentage, with larger bubbles denoting higher expression percentages

After analyzing the overall data, we found that certain key functional genes within the enriched pathways exhibited trending changes at different infection time points in unexposed cells, infected cells, and bystander cells (Supplementary Table 8). These genes play a crucial role in regulating macrophage inflammation, metabolic reaction, chemotaxis, and oxidative stress management, processes that are pivotal in the pathogenesis of leishmaniasis. Specifically, the expression percentage of *Il1rn* in the inflammation pathway in unexposed cells was 21.83%; in bystander cells at 6, 12, and 18 h, it was 60.71%, 44.00%, and 44.79%, respectively; and in infected cells at 6, 12, and 18 h, it was 64.69%, 43.73%, and 46.20%, respectively. The expression rate of this gene increased with infection. The expression percentage of *Ccl3* in unexposed cells was 61.68%; in bystander cells at 6, 12, and 18 h, it was 80.23%, 88.07%, and 89.26%, respectively; and in infected cells at 6, 12, and 18 h, it was 80.15%, 82.29%, and 83.57%, respectively. The expression rate of this gene also increased with infection. The expression percentage of *Hmox1*, which is related to oxidative stress response, in unexposed cells was 88.07%; in bystander cells at 6, 12, and 18 h, it was 97.86%, 75.67%, and 65.41%, respectively; and in infected cells at 6, 12, and 18 h, it was 96.26%, 69.89%, and 63.10%, respectively. The expression percentage of *Fabp4*, associated with metabolic reactions, in unexposed cells was 19.73%. For bystander cells, the expression percentages at 6, 12, and 18 h were 45.76%, 9.76%, and 7.58%, respectively. Infected cells showed expression percentages of 54.77%, 28.75%, and 26.82% at 6, 12, and 18 h, respectively. *Cd36* expression percentage in unexposed cells was 66.53%; in bystander cells, it was 70.84%, 47.91%, and 44.70% at 6, 12, and 18 h, respectively. Infected cells showed expression percentages of 71.91%, 52.32%, and 48.37% at 6, 12, and 18 h, respectively. *Pf4* expression in unexposed cells was 35.75%; The expression percentages in bystander cells at 6, 12, and 18 h were 29.24%, 20.78%, and 17.80%, respectively. The expression percentages in infected cells at 6, 12, and 18 h were 32.35%, 39.92%, and 40.93%, respectively (Fig. 7).

### Dynamic expression changes of parasite genes during infection

**Fig. 8:**
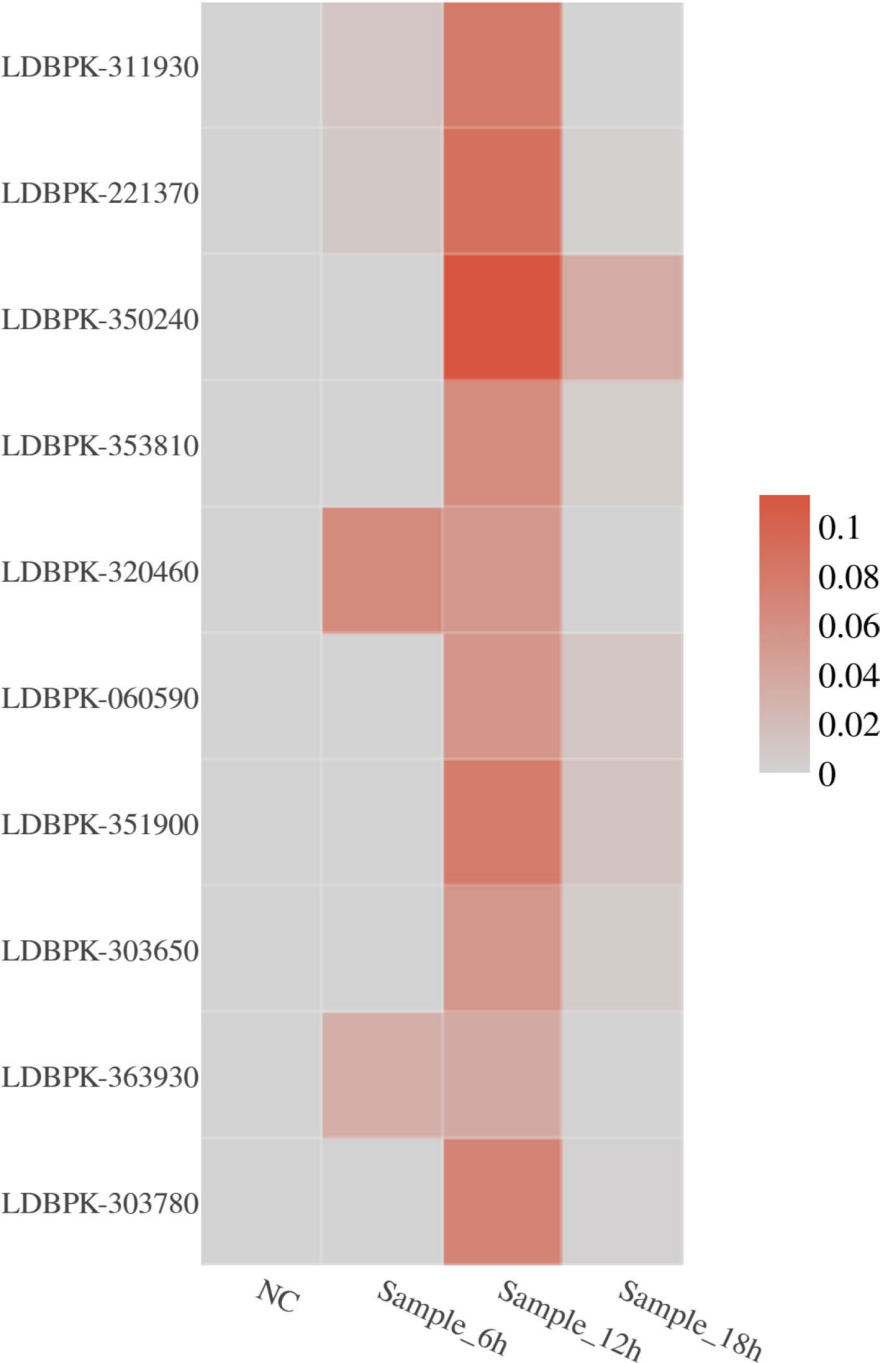
A heat map showing top-ranked parasite genes in sample cells across different time periods. The horizontal axis represents samples from four groups. The vertical axis represents individual parasite genes. The intensity of colors in the heat map indicates the expression level of parasite genes; darker colors indicate higher expression of that gene within the corresponding cell group

On the basis of parasite gene expression levels in infected cells, 2,043 parasite genes showed significant expression in the 6-h sample approximately. The top-ranked genes included LDBPK_320460, LDBPK_221370, and LDBPK_363930, all of which encode ribosomal proteins. A total of 1,256 genes were significantly expressed in the 12 h sample, with the top-ranked genes including LDBPK_350240, LDBPK_221370, and LDBPK_311930, all associated with ribosomal protein coding. A total of 1,165 genes were significantly expressed in the 18 h sample, with the top-ranked genes being LDBPK_350240, LDBPK_351900, LDBPK_060590, etc. (Fig. 8). There were 303 differentially expressed genes between the 12-h sample and 18-h sample, and 212 differentially expressed genes between the 18-h sample and 12-h sample.

### Analysis of infection rate based on cell cluster definition using M1/M2 markers

**Fig. 9:**
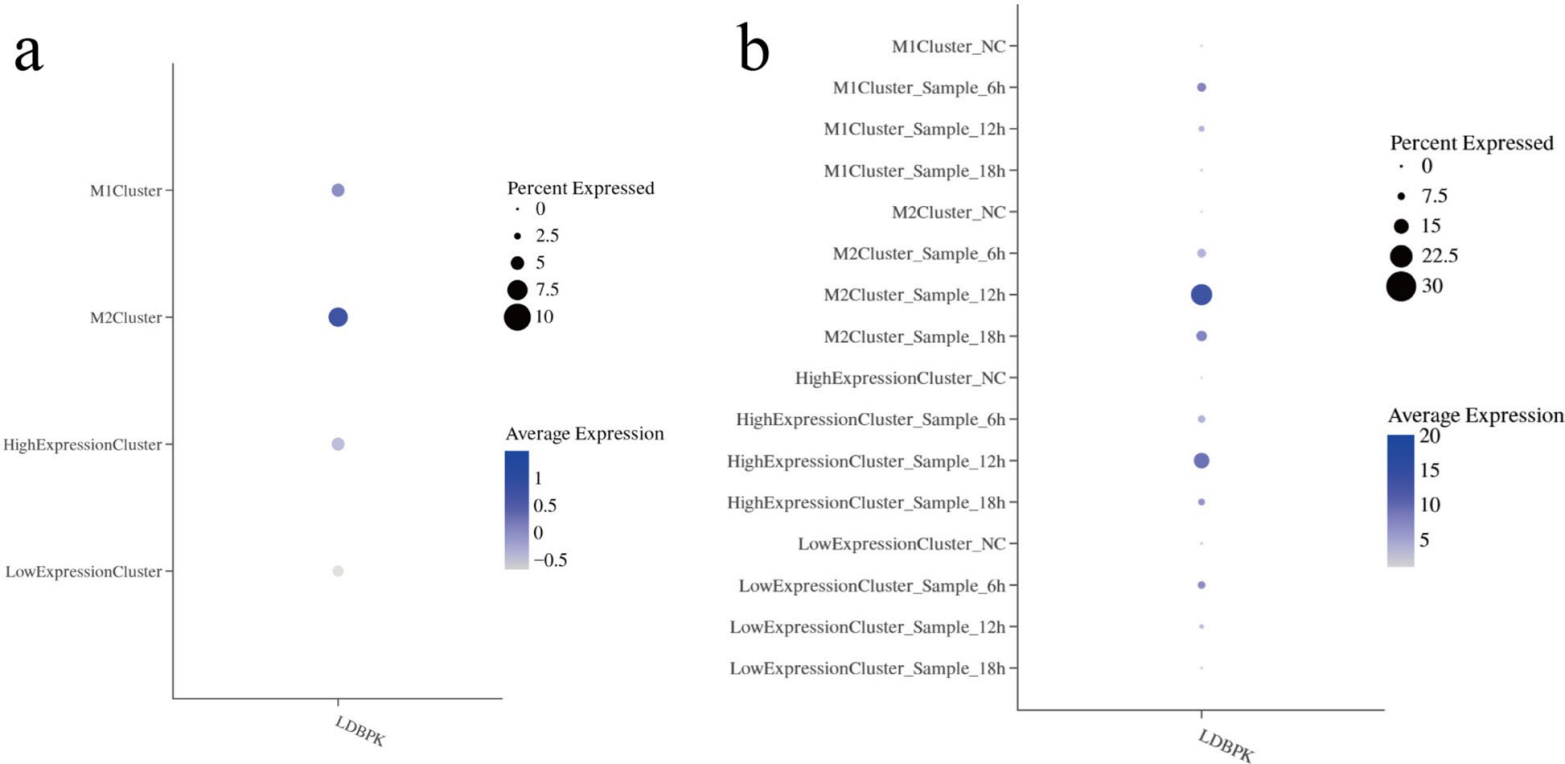
Analysis of infection rate based on cell clusters defined by M1/M2 markers. **a**, Gene expression profile of parasites in cell clusters classified based on M1/M2 markers. **b**, Parasite gene expression in cell clusters sorted on the basis of M1/M2 markers at different infection time points

We defined four cell clusters associated with polarization based on the gene expression profiles of two categories of markers: M1 markers, including *Il1a*, *Il1b*, *Il6*, *Il12rb1*, *Il12rb2*, *Il23a*, *Tnf*, *Cd68*, *Cd80*, *Cd86*, and *Irf5*, and M2 markers, including *Ido1*, *Ido2*, *Il10*, *Tgfb1*, *Tgfb2*, *Csf1r*, *Fcer1g*, *Cd163*, *Irf4*, *Stat6*, and *Cd209c*. These clusters are: M1 cluster: M1 gene expression ≥ q75 and M2 gene expression < q25; M2 cluster: M2 gene expression ≥ q75 and M1 gene expression < q25; high expression cluster: M1 gene expression ≥ q75 and M2 gene expression ≥ q75; low-expression cluster: M1 gene expression ≤ q25 and M2 gene expression ≤ q25. Subsequently, we analyzed the parasite infection rate in these four cell clusters. The results showed that the parasite gene expression percentage in the M1 cluster was 4.80%, in the M2 cluster was 7.17%, in the high expression cluster was 4.81%, and in the low expression cluster was 4.09% (Fig. 9a). Next, we analyzed parasite gene expression in the four cell clusters at different infection time points. Results showed that the percentage of parasite gene expression in the M1 cluster was 9.12%, 5.93%, and 2.85% at 6 h, 12 h, and 18 h, respectively; the percentage in the M2 cluster was 8.91%, 21.25%, and 10.58% at 6 h, 12 h, and 18 h, respectively. The parasite gene expression percentage in the high-expression cluster was 7.70%, 15.84%, and 6.73% at 6 h, 12 h, and 18 h, respectively; the parasite gene expression percentage in the low-expression cluster was 7.76%, 4.57%, and 3.40% at 6 h, 12 h, and 18 h, respectively (Fig. 9b).

### Western blot and immunofluorescence verification of key genes selected by single-cell RNA sequencing

**Fig. 10:**
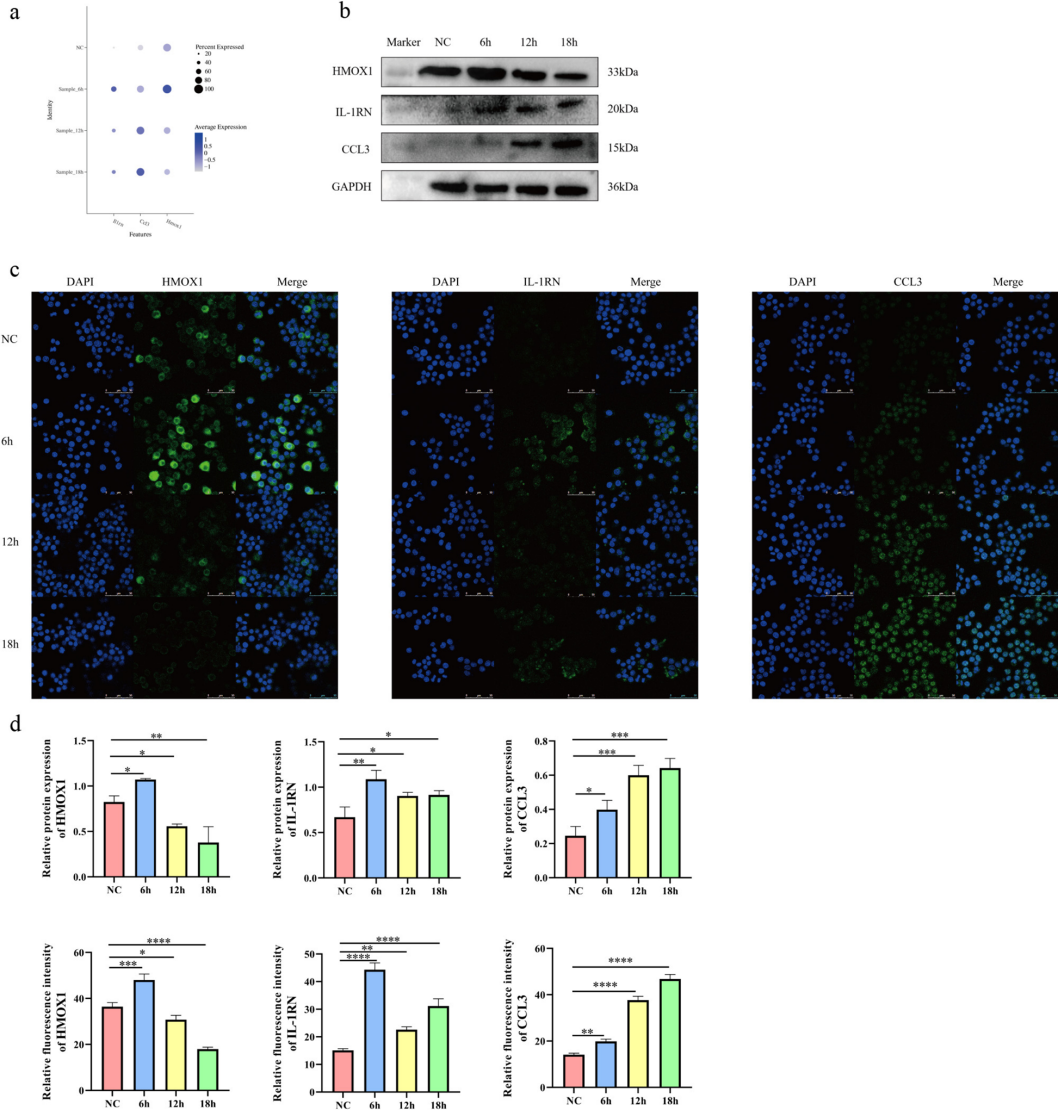
Western blot and immunofluorescence validation results. **a**, Bubble chart of scRNA-seq key gene expression. **b**, Western blot is used to verify the expression of relevant genes in the negative control group and experimental groups with different infection durations. **c**, Immunofluorescence images reveal the expression trends of relevant genes in the negative control group and experimental groups at different infection time points. DAPI is used to stain the nuclei of macrophages and *L. donovani* parasites, producing blue fluorescence; green fluorescence indicates the target protein. **d**, Western blot and immunofluorescence validation results, *P < 0.05, **P < 0.01, ***P < 0.001

We utilized western blot and immunofluorescence technology to detect three key genes: *Il1rn*, *Ccl3*, and *Hmox1* during the infection of RAW264.7 macrophages by *L. donovani*, aiming to confirm their temporal changes at the protein level. In the scRNA-seq data, they exhibited significant differential expression at three infection time points: 6 h, 12 h, and 18 h (Supplementary Table 9) (Fig. 10a). Notably, the protein expression trends of IL-1RN, CCL3, and HMOX1 were consistent with the corresponding mRNA trends observed at the same infection time points in the single-cell transcriptome data (Fig. 10b–d).

### β-Galactosidase staining validates cellular senescence pathways

**Fig. 11:**
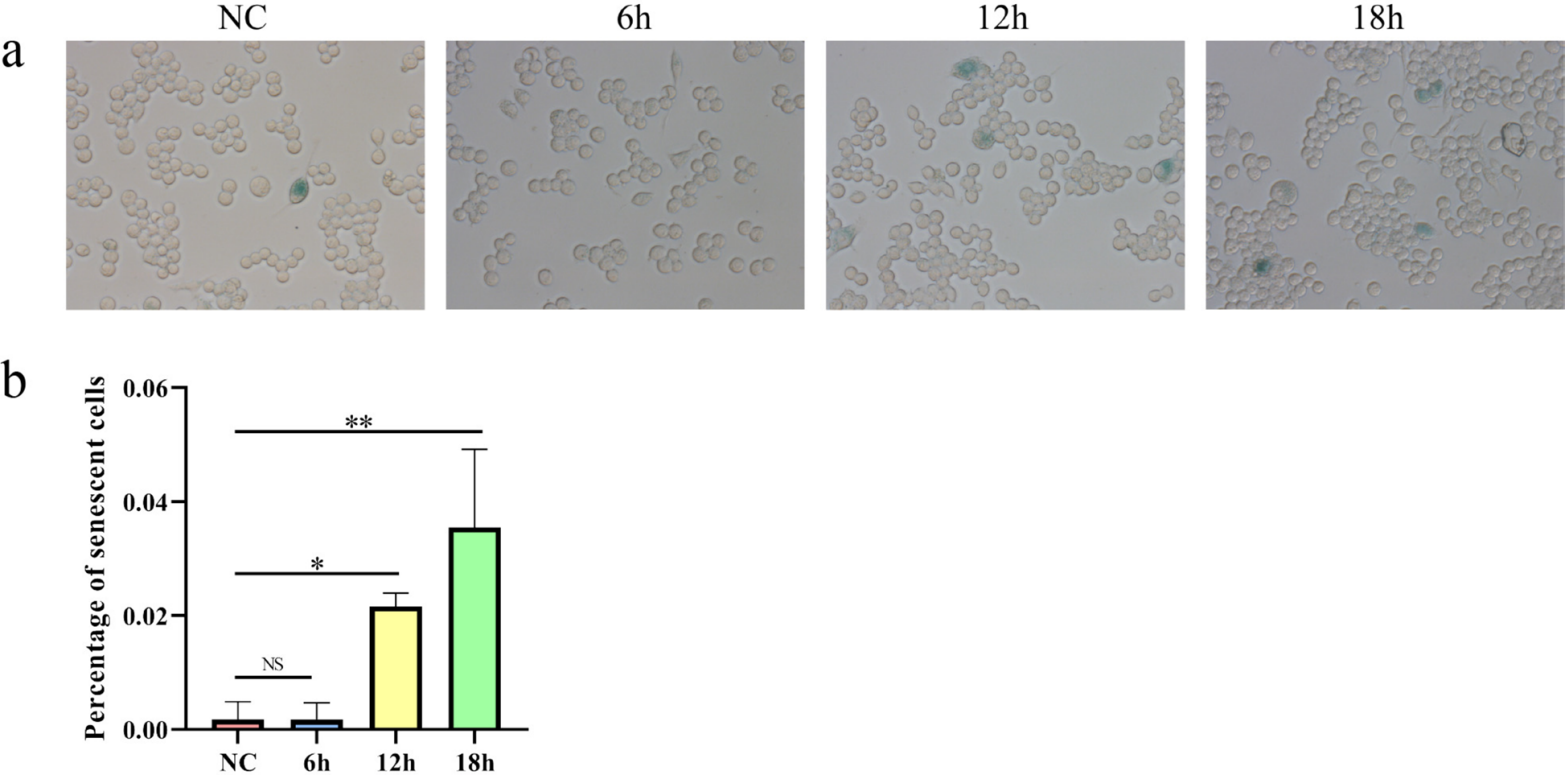
Validation of cellular aging results by β-galactosidase staining. **a**, Stain cells at different time points using a β-galactosidase staining kit, and senescent cells appear blue–green in color. **b**, The aging rate of cells in each group. NS *P* > 0.05, * *P* < 0.05, ** *P* < 0.01, *** *P* < 0.001

β-Galactosidase activity is a classic biomarker of cellular senescence, as its expression is upregulated during the aging process and lysosomal accumulation occurs, allowing detection at pH 6.0. Therefore, we validated senescence in infected and uninfected cells using β-galactosidase staining, with results shown in the figure (Fig. 11a). As infection duration increased, the number of cells stained blue progressively rose, indicating a time-dependent aging gradient consistent with mRNA expression trends observed in the single-cell transcriptomic data. Subsequently, we counted approximately 200 macrophages per group under a microscope and calculated the cellular senescence rate—the percentage of senescent cells stained blue relative to the total number of counted cells (Fig. 11b).

### *Leishmania donovani* hijacks the *Fabp4*/*Cd36* lipid metabolism pathway to promote infection

**Fig. 12:**
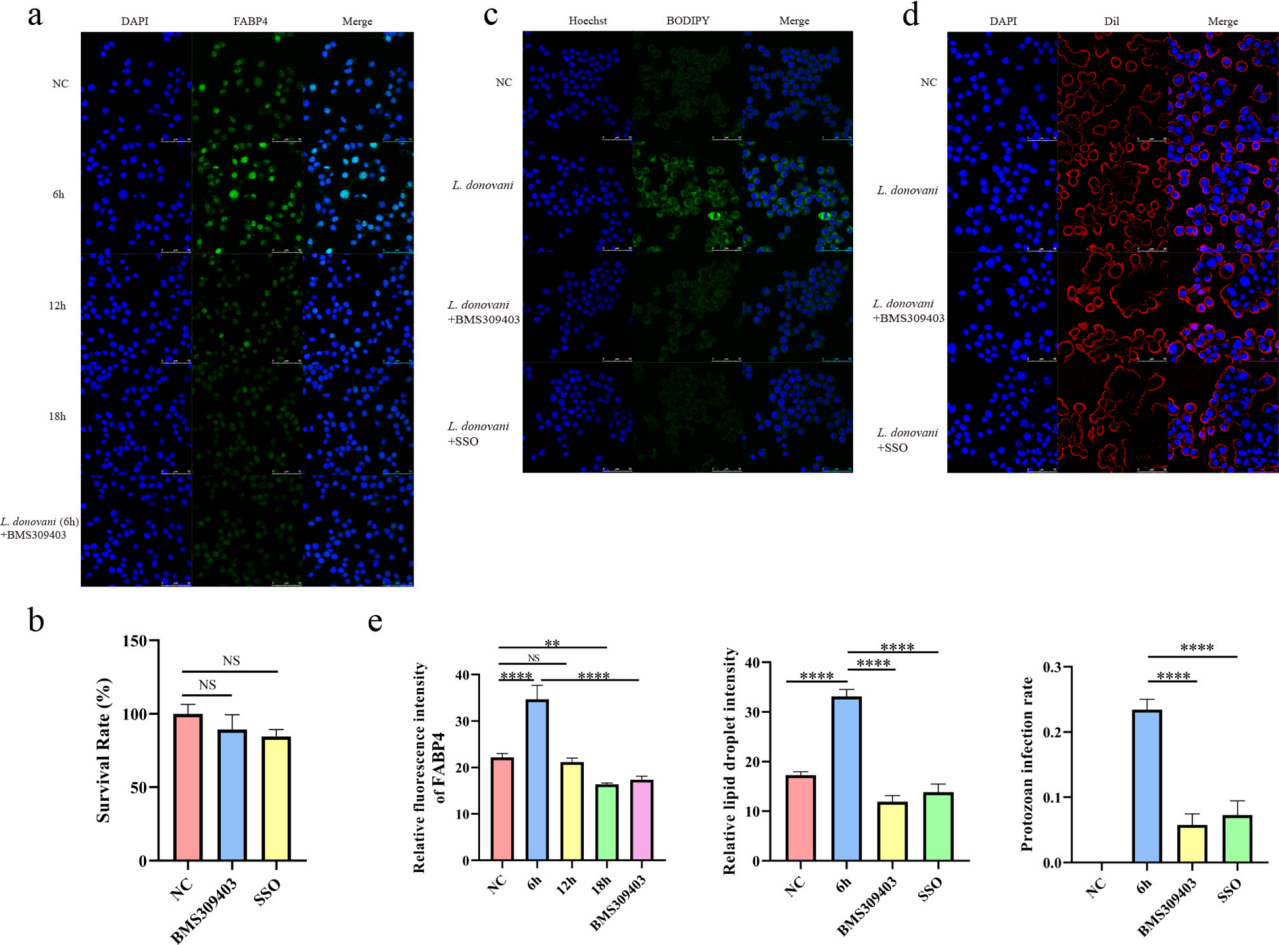
Validation results of lipid metabolism pathways. **a**, Immunofluorescence images showing FABP4 protein expression in the negative control group, experimental groups infected with parasites at 6 h, 12 h, and 18 h, and groups treated with inhibitors BMS309403 under 6-h parasite infection conditions. **b**, Cell viability assessed after 24-h pretreatment with 40 μM BMS309403 and 50 μM SSO; negative control represents synchronously cultured RAW264.7 cells. **c**, Lipid droplets stained green using a lipid droplet green fluorescence detection kit in the negative control group, the 6-h infection group, and inhibitor-treated groups under 6-h infection conditions. **d**, Parasite infection status in each group. DAPI staining shows blue fluorescence in macrophage nuclei and *L. donovani* parasite nuclei. **e**, Statistical quantitative analysis of immunofluorescence, lipid droplet staining, and infection rates. NS *P* > 0.05, *P < 0.05, **P < 0.01, ***P < 0.001

This study performed time-series immunofluorescence detection of FABP4 protein, revealing that its protein level trends were consistent with transcriptomic data (Fig. 12a). To ensure the reliability of subsequent functional assay results, this study first validated the efficacy and safety of the FABP4 inhibitor BMS309403 and the CD36 inhibitor SSO in the experimental system. First, CCK-8 assays confirmed that 24-h treatment with either inhibitor at concentrations used in subsequent functional experiments had no significant effect on the viability of RAW264.7 macrophages (Fig. 12b), ruling out interference from non-specific cytotoxicity. Second, to confirm the inhibitor’s effect on the intended target, this study examined its impact on FABP4 protein levels in parasites infected for 6 h. Immunofluorescence staining and quantitative analysis revealed that BMS309403 significantly reduced intracellular FABP4 protein expression compared with the 6-h *L. donovani*-infected group (Fig. 12a). This finding directly demonstrates that the inhibitor effectively interferes with infection-induced FABP4 upregulation. Given FABP4’s close association with lipid metabolism, this study further examined lipid droplet formation during infection. Green fluorescent lipid droplet probe staining demonstrated significantly increased lipid droplet content in cells infected for 6 h compared with the negative control group. However, pretreatment with FABP4/CD36 inhibitors markedly suppressed lipid droplet formation induced by infection at 6 h (Fig. 12c). This indicates that *L. donovani* infection promotes host cell lipid droplet accumulation during early stages by upregulating *Fabp4*. To confirm that lipid droplet formation facilitates intracellular parasite survival, we quantitatively analyzed parasite infection in different treatment groups using DAPI and Dil fluorescent staining. Under the microscope, approximately 100 macrophages were counted in each group. The infection rate (the percentage of cells containing parasites relative to the total number of cells counted) and the infection index (number of the amastigote cells in inhibitor-treated group/number of the amastigote cells in infected group × 100%) were calculated. This procedure was repeated three times. Statistical analysis revealed the highest infection rate in the 6-h infection group. By contrast, the FABP4/CD36 inhibitor-treated experimental groups exhibited significantly reduced infection rates (Fig. 12d). The infection indices for the FABP4/CD36 inhibitor-treated groups were (22.43% ± 10.66%) and (28.56% ± 6.66%), respectively, with intracellular parasites counts substantially lower than those in the 6-h infection group. The results of immunofluorescence, lipid droplet staining, and infection rates were obtained from three independent replicate experiments and subjected to statistical analysis (Fig. 12e).

## Discussion

This study focuses on *L. donovani*, one of the primary causative pathogens of visceral leishmaniasis. By applying single-cell transcriptomics to this critical host–pathogen interaction, we characterized the simultaneous changes occurring in both the invading parasite and macrophage during infection. In this study, comprehensive transcriptomic analysis of *L. donovani*-infected RAW264.7 cell samples at 6, 12, and 18 h revealed a gradual decrease in the proportion of cells expressing parasite genes over time, indicating a progressive decline in parasite infection rates within macrophages. Subsequently, we further observed the temporal changes in parasite infection rates across different cellular subpopulations. Among these, cluster 8—a distinct “high-*Fabp4*-expressing complement-associated macrophage subpopulation”—exhibited an exceptionally high infection rate during the early phase of infection (6 h). We hypothesize that infection induces high expression of FABP4, a key lipid metabolism regulator, in this subpopulation, thereby altering lipid metabolism to favor parasite colonization and survival. However, both the infection rate and FABP4 expression in this cluster significantly decreased by 12 h and remained relatively stable thereafter. This may indicate that as the infection progresses, the host’s lipid supply diminishes, further demonstrating the impact of FABP4 expression and lipid metabolism on parasite infection.

The analysis of samples over a time gradient revealed that at 6 h post parasite infection, both *Fabp4* and *Cd36* were upregulated, with the former exhibiting particularly significant elevation. FABP4 and CD36 are functionally closely coordinated lipid metabolism-related factors, responsible for transmembrane transport and intracellular shuttling of fatty acids, respectively. The upregulation of both genes synergistically enhances fatty acid transport and supply [28]. However, is this process of infection and host cell hijacking common to all exposed cells, or does it occur exclusively within infected cells? Subsequently, we further categorized the entire cell population into three groups: infected cells, bystander cells, and unexposed cells. On the basis of dynamic transcriptomic changes, we focused on comparing DEGs between infected and bystander cells. Among host genes, we observed that expression of the *Fabp4* gene in infected cells showed a significant upregulation at 6 h, followed by a return to near pre-infection levels by 12–18 h. This suggests that the parasite directly influences the metabolic state of its host cells during initial infection, establishing a lipid-rich environment conducive to its own replication. By contrast, uninfected bystander cells exhibited more pronounced *Fabp4* fluctuations. Their upregulation also peaked at 6 h, likely reflecting a transient, synchronized response to factors released by infected cells (e.g., CCL3). However, the significant downregulation observed between 12 and 18 h indicates that cells possess inhibitory regulation against abnormal lipid metabolism. This differential expression between infected and bystander cells vividly illustrates the parasite’s potent influence on macrophage lipid metabolism throughout the infected region, alongside the macrophages’ passive resistance to reduce abnormal lipid metabolism. Concurrently, *Cd36* expression showed no significant difference between infected and bystander cells, exhibiting an overall downward trend. Thus, the parasite exerts more pronounced and restrained regulation of *Fabp4* within infected cells, precisely hijacking lipid flux via this core fatty acid transporter to achieve intracellular parasitic homeostasis. Our findings also demonstrate that high-resolution techniques capable of distinguishing infected from bystander cells are essential for deepening our understanding of pathogen–host interaction mechanisms.

To validate the hypothesis that the parasite manipulates host lipid metabolism by regulating *Fabp4* and *Cd36* during the 6-h infection phase, we confirmed *Fabp4* upregulation via immunofluorescence, consistent with single-cell sequencing results. Subsequently, pretreatment of cells with FABP4/CD36 inhibitors significantly suppressed lipid droplet formation induced by 6-h infection, indicating that *L. donovani* promotes host lipid droplet accumulation during early infection by upregulating *Fabp4* and *Cd36*. Ultimately, the FABP4/CD36 inhibitor group exhibited significantly reduced parasite infection rates, confirming that *Leishmania* parasites exploit lipid droplets for survival. This synergistically expands the parasite’s “lipid refuge”, actively reshaping host metabolism to achieve “nutrient-predatory survival” (Supplementary Fig. 5). However, for *Fabp4* and *Cd36* to become novel therapeutic targets against parasite infection, further research is needed, extending from cellular studies to in vivo investigations.

Through single-cell sequencing, we captured three critical snapshots of the host–pathogen interaction. At 6 h post infection, we observed the parasite’s precise manipulation of the host’s lipid metabolism, creating a favorable environment for its survival, resulting in increased infection rates. However, at 12 and 18 h, the landscape had shifted markedly. During the late phase of *L. donovani* infection (12–18 h), lipid metabolism resembled that of uninfected samples, while aging-related genes *Plk1*, *Cenpa*, *Bub1b*, *H2afx*, and *Cdkn2d* showed upregulation, bypassing the peak infection period at 6 h. To confirm the presence of cellular senescence, β-galactosidase staining was employed in this study. The results confirmed a time-dependent increase in senescent cells, consistent with mRNA trends observed in single-cell transcriptomic data. Previous studies have found that *Plk1* upregulation abnormally phosphorylates HJURP, driving a significant increase in the centromeric protein CENPA and resulting in erroneous chromosome loading [29]. The concurrent upregulation of *Bub1b* causes spindle checkpoint failure, triggering erroneous chromosome segregation and micronucleus formation [30]. Micronucleus accumulation initiates downstream events. Chronic inflammation is mediated through the cGAS–STING pathway [31]. Persistent upregulation of *H2afx* marks DNA damage, while *Cdkn2d* induces permanent cell cycle arrest, resulting in “zombie” cells [32] (Supplementary Fig. 6)*.* Simultaneously, we observed that the initiation of cellular senescence and the decline in infection rates occurred synchronously, with the expression of senescence-related genes exhibiting the same-direction change trend in both infected and bystander cells (Supplementary Table 7). This decline in infection rates may be partly attributable to senescence-related alterations in the intracellular environment or to the persistent antimicrobial effects generated by macrophages following activation during the early stages of infection. Therefore, whether the regulation of cellular senescence pathways contributes to the reduction in infection rates will be a focus for future research.

In addition to aging-related genes exhibiting consistent trends of change in both infected cells and bystander cells, immune-related inflammatory factors *Il1rn*, *Ccl3*, and *Hmox1* also showed significant trends of change in the same direction. As an IL-1 receptor antagonist, the expression of IL-1RN significantly increased in infected cells and bystander cells, suggesting that the parasite maintains a favorable survival environment by inhibiting IL-1-mediated inflammatory responses. The expression of the chemokine *Ccl3* gene exhibited a persistent enhancement pattern, possibly indicating that the *Ccl3*-mediated recruitment of monocytes/macrophages is activated in the infected microenvironment for an extended period. Unlike inflammatory genes, the expression dynamics of the oxidative stress key gene *Hmox1* exhibited a unique trend of “early peak–late decay” in the infected microenvironment. During early infection, the parasite utilizes HMOX1 protein to decompose heme-derived iron ions to meet its own proliferation needs, while the degradation of heme can avoid the production of ROS, thereby assisting in infection colonization [33]. However, as mentioned earlier, the parasite infection rate decreases in the later stage. Therefore, we speculate that the reduced expression of *Hmox1* in the later stage may be a key defensive response of the host to inhibit parasites survival through iron restriction and restoration of reactive oxygen species (ROS) killing [34]. This was consistent with the findings of Dillon et al. in their RNA-seq study of *L. major*-infected mouse macrophages [35]. This consistency suggests that host immune inflammatory responses and the initiation of aging involves global coordination and intercellular communication within the site of infection. This may be driven by paracrine effects of soluble mediators. Upon pathogen recognition, directly infected cells release large amounts of cytokines, chemokines, and other signaling molecules. These signals diffuse into the surrounding microenvironment, activating neighboring bystander cells to synchronously upregulate key immunoregulatory genes. A summary schematic of the exploration and validation of the potential pathways is shown in Supplementary Fig. 7.

Changes in inflammatory-related factors reflect the immune regulation of host cells during *L. donovani* infection. Macrophage polarization is the core mechanism by which macrophages achieve their diverse immunoregulatory functions. Different polarization states determine macrophages’ distinct, even opposing, roles in immune responses. Macrophages can be classified into two subpopulations: classically activated (M1) and alternatively activated (M2), both of which are closely associated with inflammatory responses. Therefore, we defined cell clusters on the basis of M1/M2 markers and analyzed the potential association between different macrophage polarization states and *L. donovani* infection. Results revealed significant differences in the percentage of parasite gene expression between the M2-polarized and M1-polarized clusters among the four defined cell clusters. Furthermore, the average expression level of parasite genes in the M2 cluster was significantly higher than in the M1 cluster and other subpopulations. This aligns with classical macrophage polarization theory: M2 macrophages suppress host immune responses by secreting anti-inflammatory factors such as IL-10 and TGF-β [36], potentially providing a more favorable intracellular environment for parasite survival. Conversely, M1 macrophages primarily secrete proinflammatory cytokines such as IL-6 and IL-12 [37], exhibiting antiparasitic immune functions. Furthermore, the infection rate in the high-expression cluster was similar to that in the M1 cluster, suggesting that the coexpression of M1-associated proinflammatory genes may partially counteract the immunosuppressive effects of the M2 phenotype, establishing a dynamic equilibrium. The low-expression cluster exhibited the lowest percentage and absolute levels of parasite gene expression, potentially reflecting reduced pathogen infectivity owing to cellular quiescence or insufficient metabolic activity. Furthermore, we observed distinct temporal dynamics in this infection process. Notably, M2 macrophages exhibited the highest percentage of parasite gene expression at 12 h post infection, significantly surpassing other cell clusters. This indicates that their anti-inflammatory microenvironment is most conducive to parasite intracellular survival and proliferation. By contrast, infection rates in M1-type macrophages steadily declined over time, suggesting their proinflammatory properties may partially restrict parasite proliferation. Notably, the high-expression cluster also exhibited an infection peak at 12 h, though still lower than the M2 cluster, indicating that coexpression of M1-associated proinflammatory genes may partially interfere with the parasite’s survival advantage. Of course, studies based solely on a single cell line model are not comprehensive. Incorporating biological replicates from other macrophage lineages, such as primary cells, would enable a thorough investigation of the role of macrophage polarization in *Leishmania* infection.

This study employed time-series single-cell transcriptomics analysis to capture not only snapshots of host cells at three distinct time points post infection but also documentation of the gene expression changes in *L. donovani*. The total number of detectable parasite genes was significantly higher at 6 h than at 12 and 18 h. We hypothesize that a mixed population of promastigotes and amastigotes exists at the 6-h time point. At this stage, unengulfed promastigotes still extensively express characteristic genes (such as those related to flagellar assembly, motility, carbohydrate metabolism, and extracellular immune evasion), resulting in a high total gene detection count of 2,043. As infection progressed to 12 and 18 h, the vast majority of parasites had completed conversion to the amastigotes stage. Adapted to the acidic and nutritionally constrained intracellular environment, amastigotes undergo a metabolic shift from glycolysis-dominant to fatty acid β-oxidation-dominant metabolism. Consequently, genes nonessential for intracellular survival are largely silenced, while a core set of genes related to proliferation, stress adaptation, and host interaction are selectively retained. This results in a “streamlined and specialized” transcriptome, with the total gene count markedly reduced to approximately 1,200. Moreover, hundreds of differentially expressed genes exist between the 12-h and 18-h stages, indicating that the parasite continues to undergo transcriptional regulation to adapt to the intracellular environment even during the amastigotes phase. However, to uncover the deeper infection mechanisms of these parasites, the specific functions of their differentially expressed genes require further investigation.

## Conclusions

This study employs single-cell transcriptomics to elucidate the early dynamic interaction mechanisms between *L. donovani* and host macrophages. We reveal a core regulatory pattern: the parasite strategically hijacks the host *Fabp4*/*Cd36* lipid metabolism pathway to construct an intracellular microenvironment conducive to its survival, while simultaneously inducing a senescence-like response in infected cells. Notably, single-cell resolution analysis indicates that infection induces functional heterogeneity among host cells, with M2-like polarization correlating with higher parasite loads. Furthermore, the observed gradual decline in parasite transcriptional activity over time aligns with its developmental transitions and adaptive processes. Although based on a single cell line model, this study constructs a multidimensional mechanistic framework: *L. donovani* achieves intracellular survival through metabolic hijacking, immune regulation (evidenced by communication between infected and bystander cells), and potential senescence induction. This deep understanding of early host–pathogen dynamic interactions advances the mechanistic study of *Leishmania* infection and provides a theoretical basis for developing novel targets to block infection establishment.

## Supplementary Information

Additional file 1: Supplementary Tables 1–9 provide detailed data on sample quality control, cell cluster characteristics, infection rates, differentially expressed gene lists, pathway enrichment results, and gene expression rates across different infection time points and cell subpopulations. Supplementary Figures 1–7 present additional transcriptomic profiling, gene expression distribution, PPI networks and hub genes, lipid metabolism pathway, aging-related processes, and a summary diagram of the key pathways.

## Data Availability

The raw sequencing data generated in this study have been deposited in the NCBI Sequence Read Archive (SRA) under the BioProject accession number PRJNA1429012. The associated BioSample accessions are SAMN55977540, SAMN55977541, SAMN55977542, and SAMN55977543. All other relevant data supporting the findings of this study are available within the paper and its Additional files.

## Abbreviations

DEGs: Differentially expressed genes
VL: Visceral leishmaniasis
scRNA-seq: Single-cell RNA sequencing
PBS: Phosphate-buffered saline
FBS: Fetal bovine serum
PCA: Principal component analysis
VST: Variance-stabilizing transformation
GO: Gene ontology
KEGG: Kyoto Encyclopedia of Genes and Genomes
ORA: Over-representation analysis
PPI: Protein–protein interaction

## References

[1] Burza S, Croft SL, Boelaert M. Leishmaniasis. Lancet. 2018;392:951–70. 30126638 10.1016/S0140-6736(18)31204-2

[2] Singh OP, Hasker E, Boelaert M, Sacks D, Sundar S. Xenodiagnosis to address key questions in visceral leishmaniasis control and elimination. PLoS Negl Trop Dis. 2020;14:e0008363. 32790716 10.1371/journal.pntd.0008363 PMC7425851

[3] Ready PD. Epidemiology of visceral leishmaniasis. Clin Epidemiol. 2014;6:147–54. 24833919 10.2147/CLEP.S44267 PMC4014360

[4] van Griensven J, Diro E. Visceral leishmaniasis: recent advances in diagnostics and treatment regimens. Infect Dis Clin North Am. 2019;33:79–99. 30712769 10.1016/j.idc.2018.10.005

[5] Zaidi A, Singh KP, Anwar S, Suman SS, Equbal A, Singh K, et al. Interaction of frataxin, an iron binding protein, with IscU of Fe-S clusters biogenesis pathway and its upregulation in AmpB resistant *Leishmania donovani*. Biochimie. 2015;115:120–35. 26032732 10.1016/j.biochi.2015.05.016

[6] Afrin F, Khan I, Hemeg HA. *Leishmania*-host interactions—An epigenetic paradigm. Front Immunol. 2019;10:492. 30967861 10.3389/fimmu.2019.00492 PMC6438953

[7] Kumar D, Saha S. HAT3-mediated acetylation of PCNA precedes PCNA monoubiquitination following exposure to UV radiation in *Leishmania donovani*. Nucleic Acids Res. 2015;43:5423–41. 25948582 10.1093/nar/gkv431 PMC4477661

[8] Marr AK, MacIsaac JL, Jiang R, Airo AM, Kobor MS, McMaster WR. *Leishmania donovani* infection causes distinct epigenetic DNA methylation changes in host macrophages. PLoS Pathog. 2014;10:e1004419. 25299267 10.1371/journal.ppat.1004419 PMC4192605

[9] Liu D, Uzonna JE. The early interaction of *Leishmania* with macrophages and dendritic cells and its influence on the host immune response. Front Cell Infect Microbiol. 2012;2:83. 22919674 10.3389/fcimb.2012.00083 PMC3417671

[10] Peters NC, Khan N, Mody CH. Novel approaches to preventing phagosomal infections: timing is key. Trends Immunol. 2023;44:22–31. 36494273 10.1016/j.it.2022.11.004

[11] Lin X, Fang Y, Jin X, Zhang M, Shi K. Modulating repolarization of tumor-associated macrophages with targeted therapeutic nanoparticles as a potential strategy for cancer therapy. ACS Appl Bio Mater. 2021;4:5871–96. 35006894 10.1021/acsabm.1c00461

[12] Geissmann F, Manz MG, Jung S, Sieweke MH, Merad M, Ley K. Development of monocytes, macrophages, and dendritic cells. Science. 2010;327:656–61. 20133564 10.1126/science.1178331 PMC2887389

[13] Khandibharad S, Nimsarkar P, Singh S. Mechanobiology of immune cells: messengers, receivers and followers in leishmaniasis aiding synthetic devices. Curr Res Immunol. 2022;3:186–98. 36051499 10.1016/j.crimmu.2022.08.007 PMC9424266

[14] Loría-Cervera EN, Andrade-Narvaez F. The role of monocytes/macrophages in *Leishmania* infection: a glance at the human response. Acta Trop. 2020;207:105456. 32222362 10.1016/j.actatropica.2020.105456

[15] Gao S. Data analysis in single-cell transcriptome sequencing. Methods Mol Biol. 2018;1754:311–26. 29536451 10.1007/978-1-4939-7717-8_18

[16] Slovin S, Carissimo A, Panariello F, Grimaldi A, Bouché V, Gambardella G, et al. Single-cell RNA sequencing analysis: a step-by-step overview. Methods Mol Biol. 2021;2284:343–65. 33835452 10.1007/978-1-0716-1307-8_19

[17] Lee SH, Kang B, Kamenyeva O, Ferreira TR, Cho K, Khillan JS, et al. Dermis resident macrophages orchestrate localized ILC2 eosinophil circuitries to promote non-healing cutaneous leishmaniasis. Nat Commun. 2023;14:7852. 38030609 10.1038/s41467-023-43588-2 PMC10687111

[18] Ziegenhain C, Vieth B, Parekh S, Reinius B, Guillaumet-Adkins A, Smets M, et al. Comparative analysis of single-cell RNA sequencing methods. Mol Cell. 2017;65:631-43.e4. 28212749 10.1016/j.molcel.2017.01.023

[19] Hao Y, Hao S, Andersen-Nissen E, Mauck WM 3rd, Zheng S, Butler A, et al. Integrated analysis of multimodal single-cell data. Cell. 2021;184:3573-87.e29. 34062119 10.1016/j.cell.2021.04.048 PMC8238499

[20] Galow AM, Kussauer S, Wolfien M, Brunner RM, Goldammer T, David R, et al. Quality control in scRNA-seq can discriminate pacemaker cells: the mtRNA bias. Cell Mol Life Sci. 2021;78:6585–92. 34427691 10.1007/s00018-021-03916-5 PMC8558157

[21] Korsunsky I, Millard N, Fan J, Slowikowski K, Zhang F, Wei K, et al. Fast, sensitive and accurate integration of single-cell data with Harmony. Nat Methods. 2019;16:1289–96. 31740819 10.1038/s41592-019-0619-0 PMC6884693

[22] Yu G, Wang LG, Han Y, He QY. clusterProfiler: an R package for comparing biological themes among gene clusters. OMICS. 2012;16:284–7. 22455463 10.1089/omi.2011.0118 PMC3339379

[23] Gustavsson EK, Zhang D, Reynolds RH, Garcia-Ruiz S, Ryten M. ggtranscript: an R package for the visualization and interpretation of transcript isoforms using ggplot2. Bioinformatics. 2022;38:3844–6. 35751589 10.1093/bioinformatics/btac409 PMC9344834

[24] Wieder C, Frainay C, Poupin N, Rodríguez-Mier P, Vinson F, Cooke J, et al. Pathway analysis in metabolomics: recommendations for the use of over-representation analysis. PLoS Comput Biol. 2021;17:e1009105. 34492007 10.1371/journal.pcbi.1009105 PMC8448349

[25] Szklarczyk D, Gable AL, Nastou KC, Lyon D, Kirsch R, Pyysalo S, et al. The STRING database in 2021: customizable protein-protein networks, and functional characterization of user-uploaded gene/measurement sets. Nucleic Acids Res. 2021;49:D605–12. 33237311 10.1093/nar/gkaa1074 PMC7779004

[26] Yin L, Xiao L, Gao Y, Wang G, Gao H, Peng Y, et al. Comparative bioinformatical analysis of pancreatic head cancer and pancreatic body/tail cancer. Med Oncol. 2020;37:46. 32277286 10.1007/s12032-020-01370-0

[27] Khandibharad S, Singh S. Single-cell ATAC sequencing identifies sleepy macrophages during reciprocity of cytokines in *L. major* infection. Microbiol Spectr. 2024;12:e0347823. 38299832 10.1128/spectrum.03478-23 PMC10913457

[28] Sultana A, Rana S. Mechanisms underlying obesity-malignancy connection: a systematic narrative review. J Physiol Biochem. 2025;81:403–39. 40408050 10.1007/s13105-025-01084-9

[29] Howman EV, Fowler KJ, Newson AJ, Redward S, MacDonald AC, Kalitsis P, et al. Early disruption of centromeric chromatin organization in centromere protein A (Cenpa) null mice. Proc Natl Acad Sci U S A. 2000;97:1148–53. 10655499 10.1073/pnas.97.3.1148 PMC15551

[30] Komura K, Inamoto T, Tsujino T, Matsui Y, Konuma T, Nishimura K, et al. Increased BUB1B/BUBR1 expression contributes to aberrant DNA repair activity leading to resistance to DNA-damaging agents. Oncogene. 2021;40:6210–22. 34545188 10.1038/s41388-021-02021-y PMC8553621

[31] Chen Y, Yang C, Miao Y, Shi D, Li X, Tian S, et al. Macrophage STING signaling promotes fibrosis in benign airway stenosis via an IL6-STAT3 pathway. Nat Commun. 2025;16:289. 39753529 10.1038/s41467-024-55170-5 PMC11698984

[32] Sonzogni SV, Ogara MF, Belluscio LM, Castillo DS, Scassa ME, Cánepa ET. p19INK4d is involved in the cellular senescence mechanism contributing to heterochromatin formation. Biochim Biophys Acta. 2014;1840:2171–83. 24667034 10.1016/j.bbagen.2014.03.015

[33] Quintela-Carvalho G, Luz NF, Celes FS, Zanette DL, Andrade D, Menezes D, et al. Heme drives oxidative stress-associated cell death in human neutrophils infected with *Leishmania infantum*. Front Immunol. 2017;8:1620. 29218050 10.3389/fimmu.2017.01620 PMC5703736

[34] Saha S, Basu M, Guin S, Gupta P, Mitterstiller AM, Weiss G, et al. *Leishmania donovani* exploits macrophage heme oxygenase-1 to neutralize oxidative burst and TLR signaling-dependent host defense. J Immunol. 2019;202:827–40. 30593539 10.4049/jimmunol.1800958

[35] Dillon LA, Suresh R, Okrah K, Corrada Bravo H, Mosser DM, El-Sayed NM. Simultaneous transcriptional profiling of *Leishmania major* and its murine macrophage host cell reveals insights into host-pathogen interactions. BMC Genomics. 2015;16:1108. 26715493 10.1186/s12864-015-2237-2 PMC4696162

[36] Viola A, Munari F, Sánchez-Rodríguez R, Scolaro T, Castegna A. The metabolic signature of macrophage responses. Front Immunol. 2019;10:1462. 31333642 10.3389/fimmu.2019.01462 PMC6618143

[37] Yunna C, Mengru H, Lei W, Weidong C. Macrophage M1/M2 polarization. Eur J Pharmacol. 2020;877:173090. 32234529 10.1016/j.ejphar.2020.173090

