# Supplementary material for: Single-cell transcriptomics reveals lipid metabolism reprogramming in macrophages in vitro during early stages of Leishmania donovani infection
Source: Parasit Vectors. 2026 Mar 13;19:176. doi: 10.1186/s13071-026-07361-w (PMC13101224; doi:10.1186/s13071-026-07361-w)
Supplement: Supplementary file 1 — Additional file 1: Supplementary Tables 1–9 provide detailed data on sample quality control, cell cluster characteristics, infection rates, differentially expressed gene lists, pathway enrichment results, and gene expression rates across different infection time points and cell subpopulations. Supplementary Figures 1–7 present additional transcriptomic profiling, gene expression distribution, PPI networks and hub genes, lipid metabolism pathway, aging-related processes, and a summary diagram of the key pathways. [file 13071_2026_7361_MOESM1_ESM.pdf]

Additional file 1 of Single-cell transcriptomics reveals lipid metabolism reprogramming in macrophages in vitro during early stages of *Leishmania donovani* infection.

Supplemental Tables

Supplementary Table 1. Overview of cell analysis of samples with different infection durations

Sample	Cell_ Num	nFeature_RNA _threshold	nCount_RNA _threshold	mito_ threshold	mitorelatedgenes_ threshold	final_ num
NC	10322	343 ~ 6282	502 ~ 48753	0 ~ 20	0 ~ 20	10322
6h	10391	299 ~ 6201	500 ~ 46089	0.0596 ~ 19.6438	0.0596 ~ 19.6487	10391
12h	11205	266 ~ 5426	499 ~ 32059	0.1077 ~ 19.9679	0.1077 ~ 19.9759	11205
18h	14233	259 ~ 5800	502 ~ 36817	0.1905 ~ 20	0.1905 ~ 20	14233

Supplementary Table 2. Top 5 upregulated genes in all cell clusters across four samples

DEGs[a]	NC	6h	12h	18h
Cluster 0	*Lyz1*、*Fos*、*Cxcl2*、*Cd74*、*Junb*	*Hmox1*、*Lyz1*、*Fabp4*、*Sqstm1*、*Gclm*	*Ccl2*、*Spp1*、*Ccl4*、*Rgs2*、*Ccl9*	*Ccl2*、*Spp1*、*Ccl4*、*Ccl3*、*Ccl9*
Cluster 1	*Fos*、*Cx3cr1*、*Cks1b*、*Sdhd*、*Hdgf*	*Hmox1*、*Sqstm1*、*Gclm*、*AY036118*、*Rgs1*	*Car2*、*Cenpa*、*Ncl*、*Atp1b3*、*Hnrnpa0*	*Pttg1*、*Cenpa*、*Car2*、*Scd2*、*Cdc20*
Cluster 2	*Fos*、*Lyz2*、*Cd74*、*Pid1*、*S100a11*	*Gclm*、*Sqstm1*、*Hmox1*、*Prdx1*、*Txnrd1*	*Spp1*、*Ccl9*、*Vim*、*Ccl4*、*Ccl3*	*Spp1*、*Ccl3*、*Ccl9*、*Ccl4*、*Cdkn1a*
Cluster 3	*Hist1h1e*、*Hist1h1b*、*Lyz1*、*Mki67*、*Hist1h4d*	*Hist1h1e*、*Plau*、*Ank*、*Hist1h1b*、*Gclm*	*Ccl2*、*Ccl3*、*Ccl4*、*Rrm2*、*Spp1*	*Ccl2*、*Ccl3*、*Ccl4*、*Spp1*、*Ccl9*
Cluster 4	*Mki67*、*Hist1h4d*、*Hmgb2*、*Top2a*、*Cenpf*	*Cenpf*、*Mki67*、*Prc1*、*Top2a*、*Arl6ip1*	*Ube2c*、*Top2a*、*Nusap1*、*Aurka*、*Tubb4b*	*Ube2c*、*H2afx*、*Top2a*、*Nusap1*、*Tubb4b*

Cluster 5	*Hist1h1e*、*Hist1h1b*、*Mki67*、*Hist1h4d*、*Hist1h3c*	*Hist1h1e*、*Hist1h1b*、*Hist1h4d*、*Hist2h2ac*、*Mki67*	*Rrm2*、*Hist1h4d*、*Hist1h1b*、*Ccl4*、*Dut*	*Hist1h1b*、*Hist1h4d*、*Rrm2*、*Hist1h3c*、*Ccl4*
Cluster 6	*Fth1*、*Rpl22l1*、*Gng5*、*Rps20*、*S100a6*	*AY036118*、*Camk1d*、*Cmss1*、*Hmox1*、*Cd9*	*Gphn*、*Cmss1*、*Lars2*、*Zc3h7a*、*Spata7*	*Camk1d*、*Hist1h4d*、*AY036118*、*Ccl2*、*Cmss1*
Cluster 7	*Cenpf*、*Mki67*、*Prc1*、*Hmgb2*、*Top2a*	*Cenpf*、*Mki67*、*Prc1*、*Arl6ip1*、*Ube2c*	*Ube2c*、*Cenpf*、*Nusap1*、*Cdk1*、*Arl6ip1*	*Ube2c*、*Cenpf*、*H2afx*、*Nusap1*、*Cenpa*
Cluster 8	*C1qa*、*C1qc*、*Pf4*、*C1qb*、*Spp1*	*Fabp4*、*Hmox1*、*Pf4*、*C1qa*、*C1qb*	*C1qa*、*Spp1*、*C1qc*、*Pf4*、*Ccl12*	*Spp1*、*C1qa*、*Pf4*、*Ccl12*、*C1qc*
Cluster 9	*Fth1*、*Rps20*、*Rps4x*、*Gng5*、*Atox1*	*Fth1*、*Ftl1*、*Bri3*、*Gm10076*、*Ost4*	*Spata7*、*Rps29*、*Ccl4*、*Rps21*、*Hk2*	*Rps29*、*Spata7*、*Rps21*、*Hk2*、*Rps28*
Cluster 10	*Malat1*、*mt-Nd5*、*Pde4d*、*Dock10*、*Lrmda*	*Cmss1*、*mt-Nd4l*、*Camk1d*、*mt-Nd5*、*Rgs1*	*Malat1*、*mt-Atp8*、*Gphn*、*mt-Nd5*、*mt-Co1*	*Malat1*、*mt-Nd5*、*Nfat5*、*Pde4d*、*mt-Atp8*
Cluster 11	*Ccl5*、*Isg15*、*Ifi202b*、*Ifi204*、*Phf11d*	*Isg15*、*Oasl1*、*Ccl5*、*Rsad2*、*Ifi202b*	*Isg15*、*Cxcl10*、*Ifi202b*、*Ccl4*、*Ifi44*	*Cxcl10*、*Isg15*、*Rsad2*、*Ifi202b*、*Ccl4*
Cluster 12	*Lyz1*、*Itga4*、*Spp1*、*Ctsh*、*Cxcl2*	*Lyz1*、*Hmox1*、*Gclm*、*Acod1*、*Spp1*		

a Differentially expressed genes

Supplementary Table 3. Infection rate of cell clusters with parasite in samples with different infection durations

Cell cluster	6 hour	12 hour	18 hour
Cluster 0	6.64%	10.71%	8.29%
Cluster 1	3.99%	2.17%	2.04%
Cluster 2	6.98%	5.93%	3.60%
Cluster 3	7.14%	9.61%	6.38%
Cluster 4	8.00%	2.32%	2.36%
Cluster 5	4.95%	3.55%	2.00%
Cluster 6	13.76%	11.52%	4.65%
Cluster 7	3.28%	2.32%	1.11%
Cluster 8	51.06%	29.70%	29.09%
Cluster 9	5.19%	1.57%	0.91%
Cluster 10	13.75%	29.81%	24.69%
Cluster 11	10.20%	4.19%	2.48%
Cluster 12	1.43%	0	

Supplementary Table 4. Analysis of gene expression differences in macrophages at different infection times

DEGs	6hVSNC	12hVSNC	18hVSNC	12hVS6h	18hVS6h	18hVS12h
DEGs number	355	1108	1136	1421	1464	17
Top 10 up-regulated gene	*Hmox1*、*Il1rn*、*Plau*、*Actg1*、*Fabp4*、*Ccl3*、*Gclm*、*Sqstm1*、*Ctnnb1*、*Txnrd1*	*Ccl4*、*Ccl3*、*Spata7*、*Car2*、*Spp1*、*Ccl9*、*Ube2c*、*Lpl*、*Hk2*、*Actg1*	*Ccl4*、*Ccl3*、*Spp1*、*Ccl9*、*Car2*、*Ube2c*、*Spata7*、*Ccl2*、*Lpl*、*Cenpa*	*Ccl4*、*Car2*、*Ube2c*、*Pclaf*、*Ly6e*、*Spp1*、*Dut*、*Selenoh*、*Cenpa*、*Hmgb2*	*Ccl4*、*Spp1*、*Car2*、*Ube2c*、*Pclaf*、*Ifitm3*、*Ccl9*、*Ly6e*、*Cenpa*、*Pttg1*	*Spp1*、*H2afx*、*Fdps*、*Hspa5*、*Ifitm3*、*Scd2*、*Ldlr*、*Cox8a*

Top 10 down-regulated gene	*Hmgb2*、*Pid1*、*Dut*、*Fos*、*Mthfd2*、*Ab124611*、*Gmnn*、*Cx3cr1*、*Pclaf*、*Ai506816*	*Lyz1*、*Cd74*、*Cxcl2*、*Fos*、*Rgs1*、*Gclm*、*Dusp1*、*Sqstm1*、*Fth1*、*Creg1*	*Lyz1*、*Fos*、*Cd74*、*Cxcl2*、*Gclm*、*Rgs1*、*Fth1*、*Sqstm1*、*Creg1*、*Dusp1*	*Lyz1*、*Gclm*、*Hmox1*、*Sqstm1*、*Cxcl2*、*Cd74*、*Rgs1*、*Ank*、*Txnrd1*、*Clec4e*	*Hmox1*、*Gclm*、*Lyz1*、*Sqstm1*、*Txnrd1*、*Ank*、*Rgs1*、*Clec4e*、*Cxcl2*、*Cd74*	*Plin2*、*Hmox1*、*Glrx*、*Ld1*、*C1qa*、*Micos10*、*Ld3*、*Rab3gap1*、*C1qc*

Supplementary Table 5. Overview of gene expression differences in related pathway

Gene	6h VS NC(avg_log_2FC)	12h VS NC(avg_log_2FC)	18h VS NC(avg_log_2FC)
Il1rn	1.147	0.796	0.896
Ccl3	1.065	1.872	2.128
Hmox1	1.731	-0.467	-0.903
Fabp4	1.069	-0.136(NS)	-0.271
Cd36	0.328	-0.446	-0.563
Plk1	-0.051(NS)	0.413	0.493
Cenpa	-0.134(NS)	1.144	1.245
Bub1b	-0.158(NS)	0.395	0.456
H2afx	-0.344	0.627	0.986
Cdkn2d	-0.134(NS)	0.171(NS)	0.284

Supplementary Table 6. Differential gene expression analysis in infected cells and bystander cells at different infection times

DEGs	6h	12h	18h
DEGs number	17	193	175
Top 20 up-regulated gene	LDBPK_320460、<br>LDBPK_221370、<br>LDBPK_350240、<br>LDBPK_353330、<br>LDBPK_260160、<br>LDBPK_363930、<br>LDBPK_353810、<br>LDBPK_060590、	LDBPK_350240、<br>LDBPK_221370、<br>LDBPK_320460、<br>LDBPK_351900、<br>LDBPK_353810、<br>LDBPK_060590、<br>LDBPK_303780、<br>LDBPK_303710、	LDBPK_350240、<br>LDBPK_221370、<br>LDBPK_351900、<br>LDBPK_320460、<br>LDBPK_353810、<br>LDBPK_060590、<br>LDBPK_303780、<br>LDBPK_300690、

	LDBPK_111110、LDBPK_220004、LDBPK_212090、LDBPK_333300、LDBPK_311930、*Fabp4*、*Lars2*、*Fabp5*	LDBPK_210800、LDBPK_260160、LDBPK_311930、*Lars2*、*mt-Co1*、*Fabp4*、*Gpnmb*、*Spp1*、*C1qa*、*mt-Co2*、*mt-Co3*、*mt-Atp6*	LDBPK_311930、*Fabp4*、*Lars2*、*mt-Co1*、*Pf4*、*C1qa*、*Gpnmb*、*Osbpl8*、*mt-Co2*、*mt-Co3*、*Spp1*、*C1qc*
Top 10 down-regulated gene	*Cx3cr1*	*Lpl*、*Ncl*、*Set*、*Rps7*、*Ptma*、*Atp1b3*、*Snrpb2*、*Ssb*、*Rps21*、*Hspd1*	*Lpl*、*Set*、*Rhox5*、*Ifi27*、*Chchd10*、*Dut*、*Rpl7*、*Rps7*、*Ncl*、*Ptma*

Supplementary Table 7. Overview of differentially expressed genes between infected and bystander cells in related pathway at different infection time points

Gene	6h infected cell VS bystander cell (avg_log$_2$FC)	12h infected cell VS bystander cell (avg_log$_2$FC)	18h infected cell VS bystander cell (avg_log$_2$FC)
Il1rn	0.346(NS)	0.126(NS)	0.130(NS)
Ccl3	-0.103(NS)	0.192(NS)	0.181(NS)
Hmox1	0.094(NS)	0.294(NS)	0.233(NS)
Fabp4	0.331	0.970	1.034
Cd36	0.171(NS)	0.365(NS)	0.369(NS)
Plk1	-0.008(NS)	-0.142(NS)	-0.031(NS)
Cenpa	-0.046(NS)	-0.228(NS)	-0.032(NS)
Bub1b	-0.006(NS)	-0.186(NS)	-0.138(NS)
H2afx	-0.064(NS)	0.006(NS)	-0.180(NS)
Cdkn2d	-0.008(NS)	-0.042(NS)	-0.061(NS)

Supplementary Table 8. Partial gene expression rates of cell subpopulations at different infection time gradients

Gene	*Il1rn*			*Ccl3*			*Hmox1*			*Fabp4*			*Cd36*			*Pf4*		
	6h	12h	18h	6h	12h	18h	6h	12h	18h	6h	12h	18h	6h	12h	18h	6h	12h	18h
infected cell	64.69%	43.73%	46.20%	80.15%	82.29%	83.57%	96.26%	69.89%	63.10%	54.77%	28.75%	26.82%	71.91%	52.32%	48.37%	32.35%	39.92%	40.93%

bystander cell	60.71%	44.00%	44.79%	80.23%	88.07%	89.26%	97.86%	75.67%	65.41%	45.76%	9.76%	7.58%	70.84%	47.91%	44.70%	29.24%	20.78%	17.80%
unexposed cell		21.83%			61.68%			88.07%			19.73%			66.53%			35.75%	

Supplementary Table 9. Key gene expression rates in scRNA-seq at different infection times

Gene	NC	6h	12h	18h
Il1rn	21.83%	61.00%	43.98%	44.85%
Ccl3	61.68%	80.22%	87.69%	89.00%
Hmox1	88.07%	97.74%	75.29%	65.31%

Supplemental Figures

Supplementary Figure 1. Transcriptomic profiling and functional enrichment analysis of differentially expressed genes.

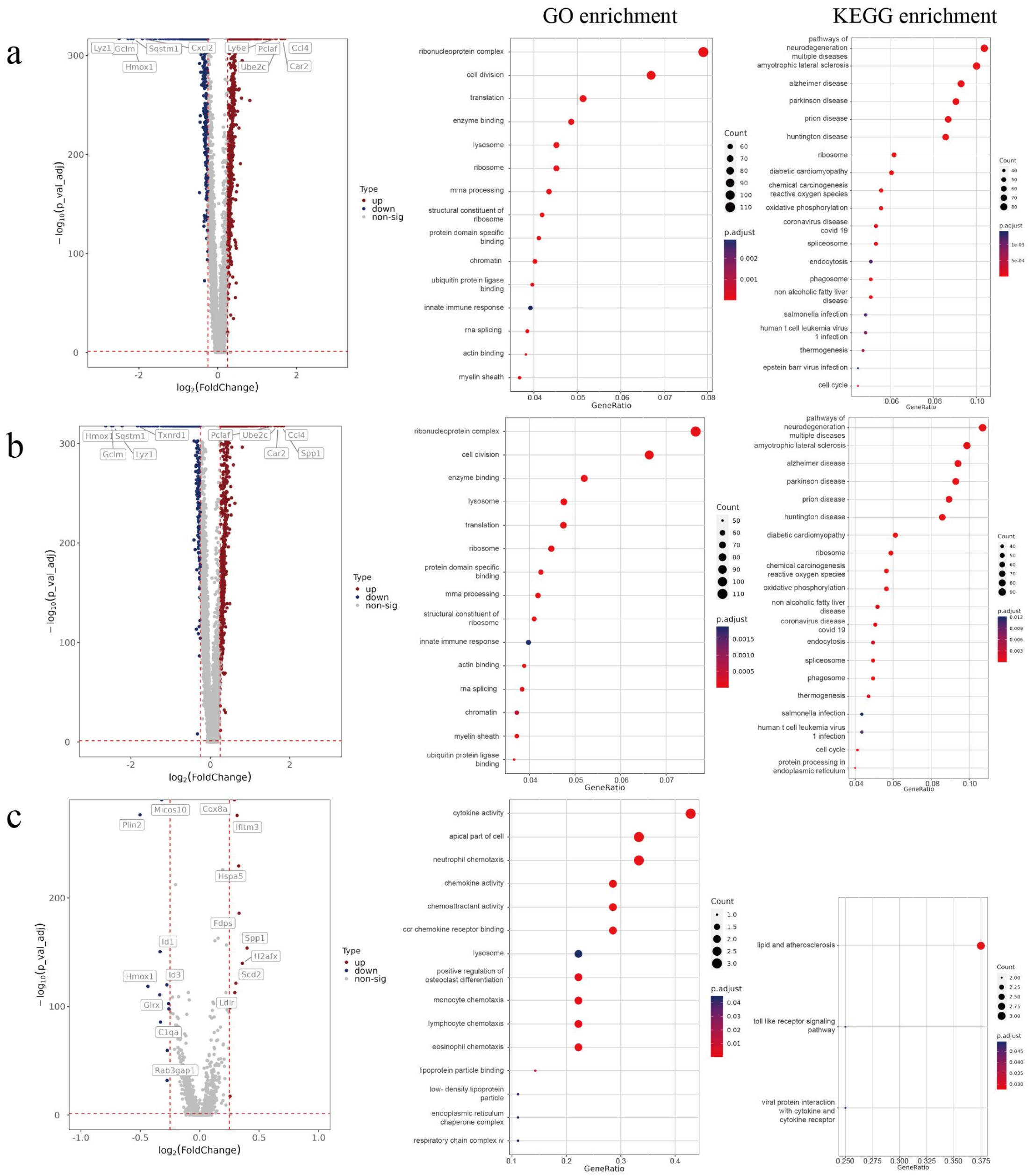

a-c. Comparative analysis between time points: Volcano plots and GO/KEGG enrichment bubble plots of DEGs for (a) 12h vs. 6h, (b) 18h vs. 6h, and (c) 18h vs. 12h sample groups.

Supplementary Figure 2. Analysis of gene expression differences among samples.

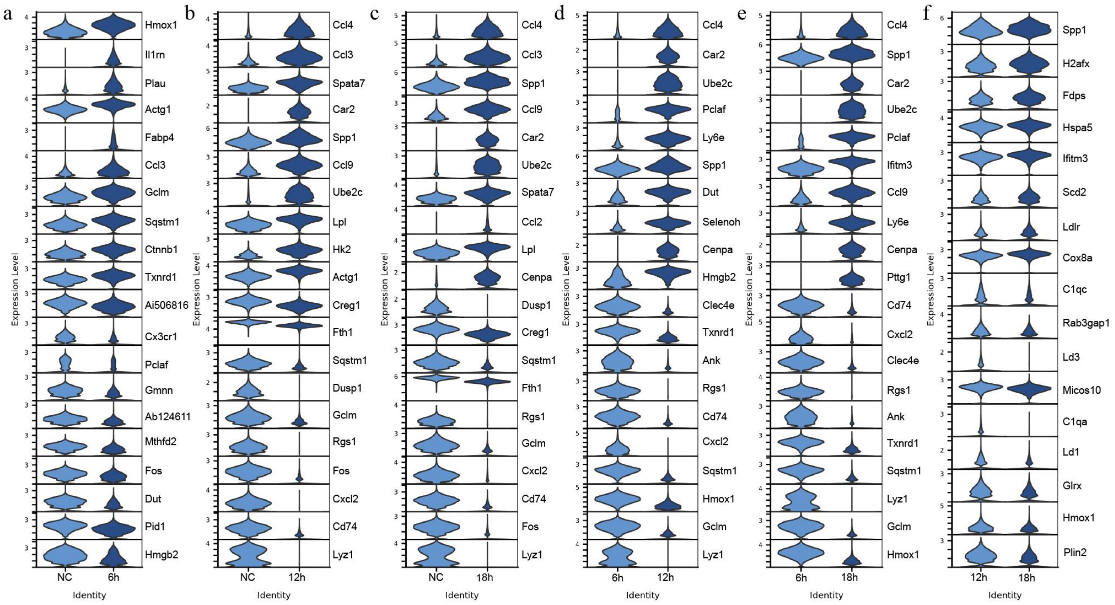

The violin plot displays the top ten up-regulated and top ten down-regulated differentially expressed genes identified through pairwise comparisons among all samples. a. 6h vs. NC. b. 12h vs. NC. c. 18h vs. NC. d. 12h vs. 6h. e. 18h vs. 6h. f. 18h vs. 12h.

Supplementary Figure 3. Expression distribution of differentially expressed genes across the entire cell.

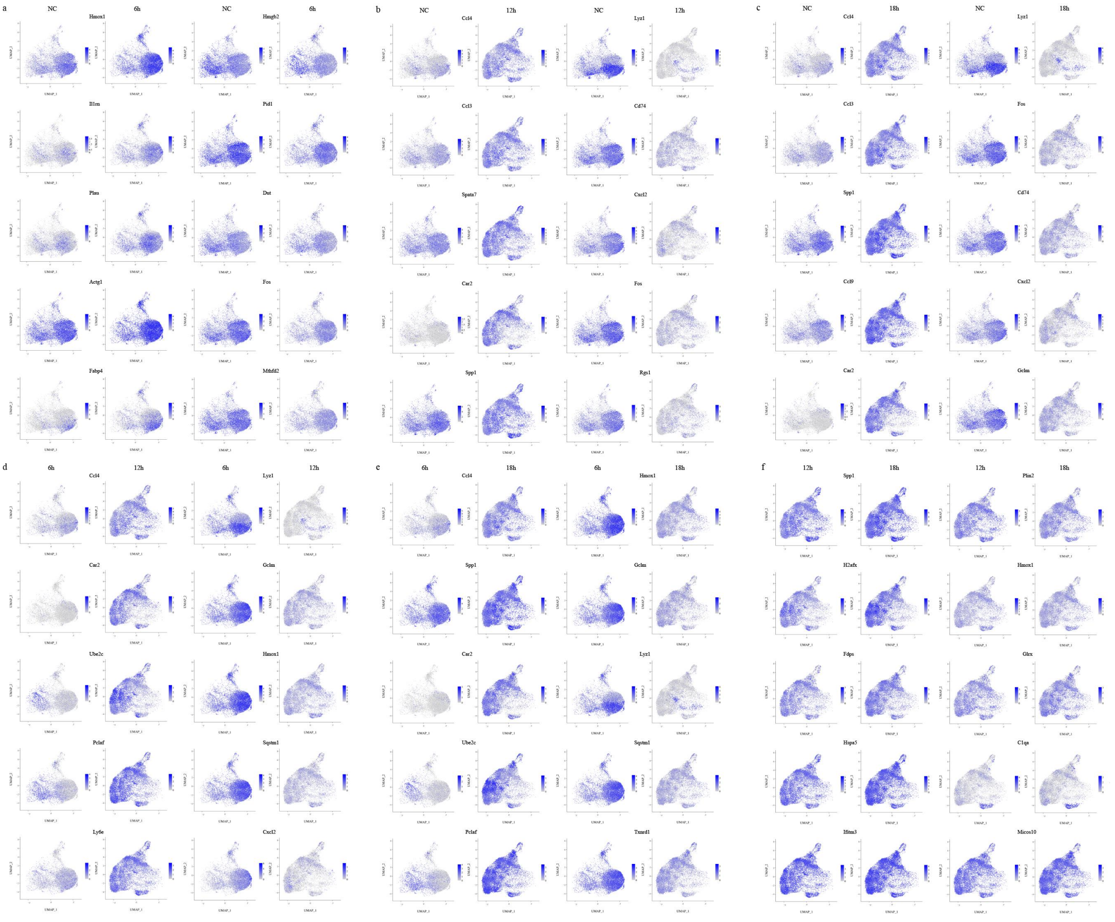

Map the expression levels of the top 5 upregulated genes and top 5 downregulated differentially expressed genes identified from pairwise comparisons across all samples onto the UMAP plot. a. 6h vs. NC. b. 12h vs. NC. c. 18h vs. NC. d. 12h vs. 6h. e. 18h vs. 6h. f. 18h vs. 12h.

Supplementary Figure 4. Protein-protein interaction networks and hub genes of top differentially expressed genes.

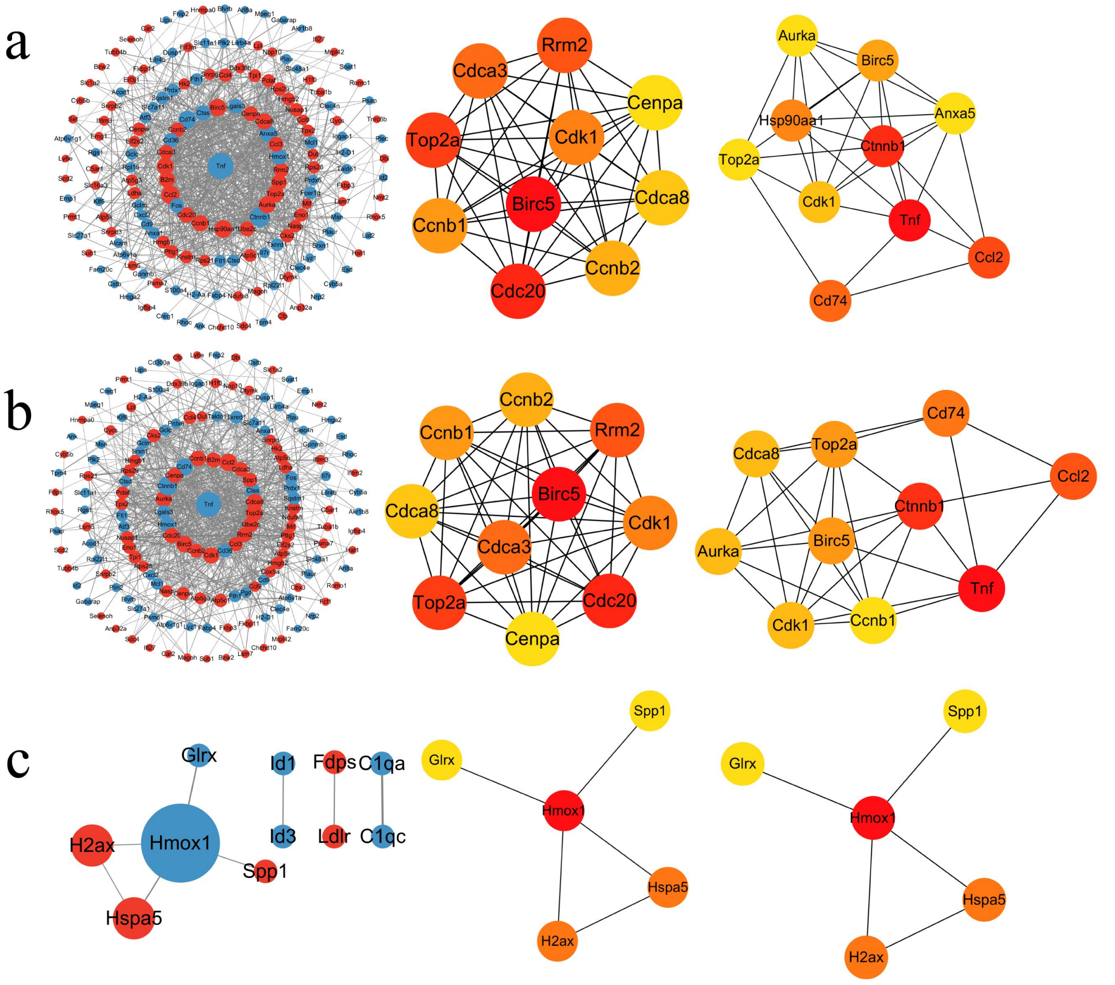

PPI networks were constructed using the top 200 DEGs from each pairwise comparison. Within each network, node colors indicate differential expression: red for upregulated and blue for downregulated genes. Hub genes within each network were identified using the CytoHubba plugin for Cytoscape. From left to right: PPI networks, top ten hub genes identified by MCC and Degree algorithms. a. 12h vs. 6h. b. 18h vs. 6h. c. 18h vs. 12h.

Supplementary Figure 5. *Fabp4/Cd36* lipid metabolism pathway.

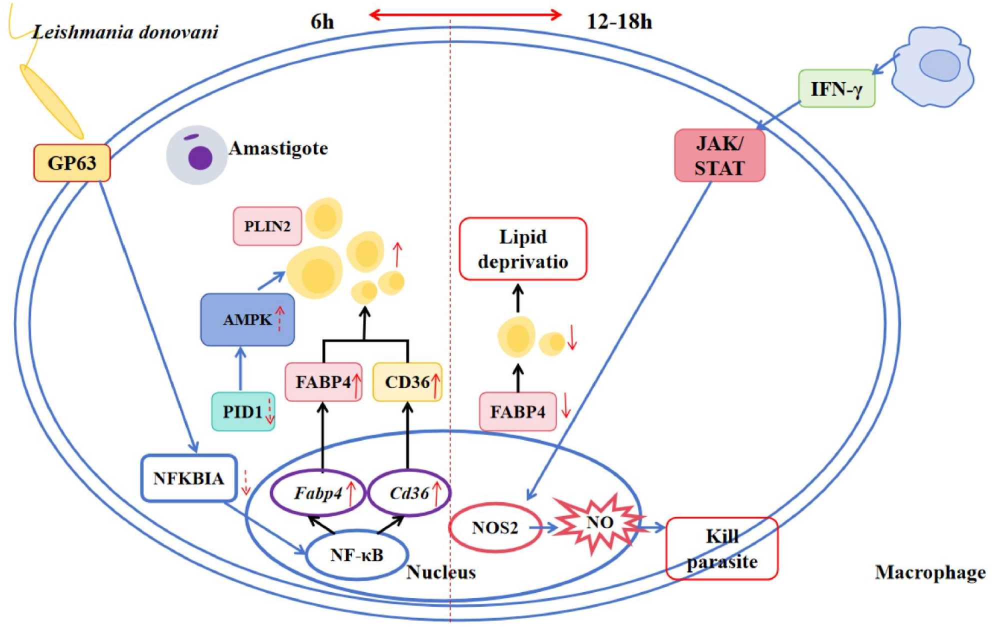

The black lines in the figure represent the experimental verification process, while the blue lines indicate the prediction process. The solid red arrows denote the actual up-regulated and down-regulated results based on experimental results, and the dashed arrows represent inferred outcomes.

Supplementary Figure 6. Aging-related processes.

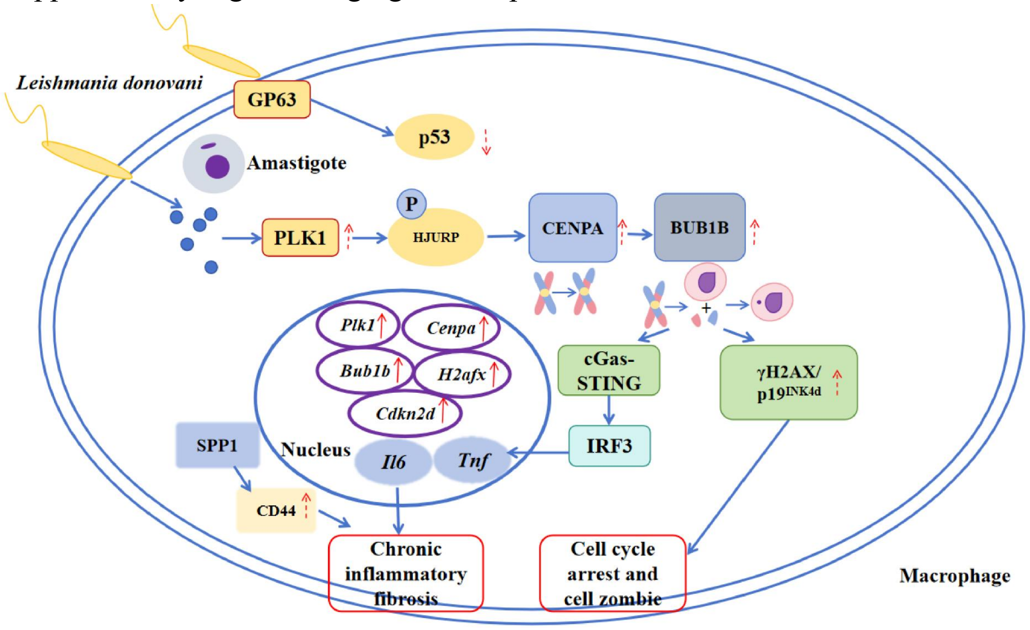

The black lines in the figure represent the experimental verification process, while the blue lines indicate the prediction process. The solid red arrows denote the actual up-regulated and down-regulated results based on experimental results, and the dashed arrows represent inferred outcomes.

Supplementary Figure 7. Summary diagram of the pathway.

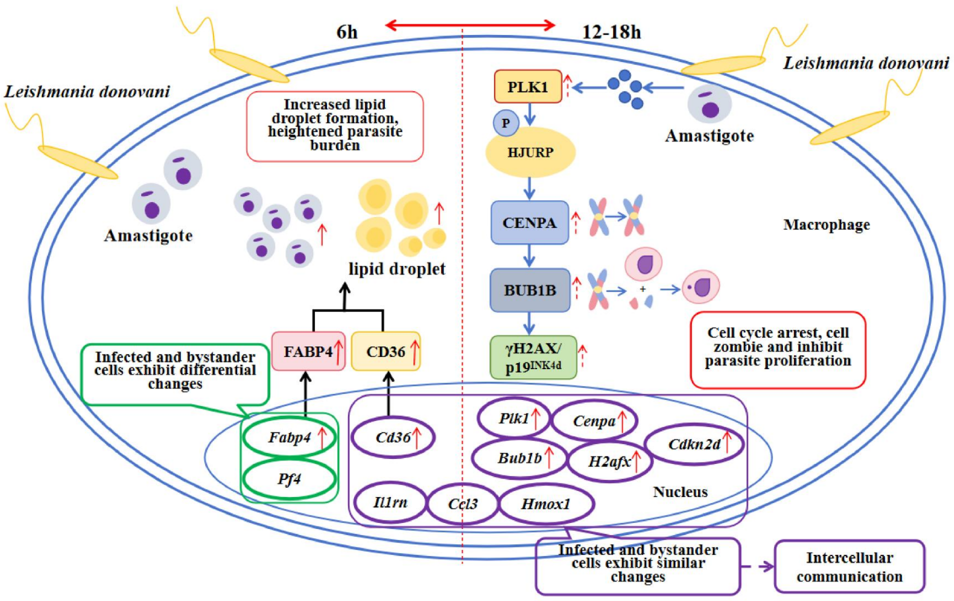

The black lines in the figure represent the experimental verification process, while the blue lines indicate the prediction process. The solid red arrows denote the actual up-regulated and down-regulated results based on experimental results, and the dashed arrows represent inferred outcomes.
